# Acrylate and Methacrylate Polymers’ Applications: Second Life with Inexpensive and Sustainable Recycling Approaches

**DOI:** 10.3390/ma15010282

**Published:** 2021-12-31

**Authors:** Carmelo Corsaro, Giulia Neri, Antonio Santoro, Enza Fazio

**Affiliations:** 1 Department of Mathematical and Computational Sciences, Physics Science and Earth Science, University of Messina, 98166 Messina, Italy; ccorsaro@unime.it; 2 Department of Chemical, Biological, Pharmaceutical and Environmental Sciences, University of Messina, 98166 Messina, Italy; giulia.neri@unime.it (G.N.); antonio.santoro@unime.it (A.S.)

**Keywords:** acrylate and methacrylate polymers, polyelectrolytes, polymer functionalization, biomedicine, environmental remediation, food packaging, electronic devices, recyclability

## Abstract

Polymers are widely employed in several fields thanks to their wide versatility and the easy derivatization routes. However, a wide range of commercial polymers suffer from limited use on a large scale due to their inert nature. Nowadays, acrylate and methacrylate polymers, which are respectively derivatives of acrylic or methacrylic acid, are among the most proposed materials for their useful characteristics like good biocompatibility, capping ability toward metal clusters, low price, potentially recyclability and reusability. Here, we discuss the advantages and challenges of this class of smart polymers focusing our attention on their current technological applications in medical, electronic, food packaging and environmental remediation fields. Furthermore, we deal with the main issue of their recyclability, considering that the current commercial bioplastics are not yet able to meet the global needs as much as to totally replace fossil-fuel-based products. Finally, the most accredited strategies to reach recyclable composites based on acrylic polymers are described.

## 1. Introduction

The design and development of new, highly efficient systems for food industry [1,2], electronic [3,4], information science [5,6] biomedicine [7,8] and environmental [9,10] applications constitute an important challenge. For such ambitious purpose it is mandatory to choose ecofriendly materials or recyclable ones, showing wide versatility in terms of structure and physicochemical properties [11,12].

Polymers represent one of the most promising candidates thanks to their high strength-to-weight ratio, stiffness, toughness, ductility and cost/effectiveness [13,14,15,16]. Moreover, they have a wide range of operating temperatures, high thermal/electrical insulation, good resistance to both corrosion and light. Furthermore, their relatively easy derivatization allows for a fine tuning of their properties [17,18]. Polymers can be synthetized employing several starting materials from petroleum to non-fossil ones. Bio-based compounds with unique chemical functionality can be obtained through selective transformations of plant and other non-fossil, biogenic feedstocks. Although substantial efforts have been made to replace polymers produced by fossil sources with ones made by green starting materials, the target has not been still reached [19,20]. 

Mechanical and thermal properties as well as porosity and wettability constitute the most important physical parameters to consider when designing the desired/required system. Different strategies are used to improve specific features of the polymeric materials for being responsive to optical, magnetic, electronic and biological effects [21,22]. Among them, the molecular functionalization in the micro- and nano-scale range was widely used to modify polymers morphology (size, shape etc), surface chemistry [23]. On the other hand, biocompatibility and biodegradation are fundamental properties for eco-friendly products, both to improve human life (i.e., scaffolds for tissue engineering) and for environmental preservation (i.e., suitable bio-package) [24].

Among the polymers family, polyelectrolytes are versatile materials characterized by a high amount of ionizable groups. Compared to uncharged polymers, polyelectrolytes possess relevant water solubility, capability to swell and form hydrogel by retention of a large amounts of water molecules and due to the ability to establish strong interactions with opposite charged macromolecules or surface [25]. Thanks to these properties, polyelectrolytes are largely employed as rheology and surface modifiers [26]. In addition, flat surfaces functionalized with polyelectrolyte multilayers can act as antimicrobial surfaces or as supports for tissue engineering [27,28]. These kinds of polymers are used in ceramic slurries as stabilizing agents [29], in concrete mixtures as superplasticizers [30] and in water treatment as flocculation agents [31]. The ability to interact at the atomic level with various types of active compounds made them suitable active colloidal supports for loading/release of drugs, proteins, enzymes, or pollutants as dyes, pesticides, humic acids and heavy metal ions [27,32]. Certainly, their properties are strongly correlated to the electrostatic interactions established in the polyelectrolyte solutions and thus depend on the pH value and ionic strength of the solution [25]. 

Polyacrylates and polymethacrylates derive from acrylic acid or methacrylic acid respectively; among them the most famous is poly(methyl methacrylate) (PMMA). The physicochemical properties of these materials strongly depend on the nature of the substituents in the side chain, while the polymerization occurs by vinyl group. The interest about them is due to their light transparency, elasticity, good weatherability and resilience, together with easy functionalization route, good biocompatibility and low cost [33]. Moreover, the poly(acrylic acid) and poly(methacrylic acid) (PAA and PMAA) and their corresponding salts are anionic polyelectrolytes with good hydrophilicity and greater ability to swell compared to the neutral polyacrylates or methacrylates [34,35]. All these features lead to the employment of these polymers in several fields from biomedicine to food industry, to environmental remediation and electronic applications. In the latter field, hybrid polyelectrolyte multilayers, obtained by layer-by-layer techniques, show the best performance in terms of sensor selectivity and sensitivity [27,36].

On one hand the long life of these synthetic polymers represents a great advantage, for instance in dental or cranioplasty implants [37]; on the other hand, their non-biodegradability is an issue with relevant environmental impact. To address this issue, modifications of the polymer structure or high performing recycle processes represent two valid ways to follow. 

In this review, we first give a summary on the relationship between chemical structure and physical properties of acrylate or methacrylate polymer-based products. Then, we deeply investigate the recent applications of poly(acrylic acid) and poly(methacrylic acid) and of their corresponding sodium salts and methyl ester derivatives in a wide range of fields from biomedicine to food packaging, electronic, water treatment and, generally, for products and devices useful to improve human life and to ensure environmental protection.

## 2. Acrylate and Methacrylate Polymers: Structure and Properties

Polyacrylate and polymethacrylate are two families of differently functionalized vinyl polymers, constituted by acrylate or methacrylate monomers in which the vinyl group is involved in polymerization reactions, while the carboxyl group can be the target of a wide range of derivatization reaction. The chemical nature of the substituents in the side chain of the polymers plays a key role in the obtainment of the desired physicochemical properties [33]. Poly(methacrylic acid) (PMAA) was the first methacrylic polymer to be synthesized in 1880 while its methyl ester derivative, called poly(methyl methacrylate) (PMMA), which is nowadays the most employed methacrylate polymer, was obtained for the first time in 1928 and marketed in 1933 [38]. Since then, the derivatization of PMAA with several functional groups leaded to a wide variety of poly(n-alkyl acrylates) such as polyethyl, polybutyl, polyhydroxyethyl, polyhydroxybutyl acrylates and so on. 

In a close relation with polymethacrylates, it is worth mentioning polyacrylates, which came from the lack of the methyl groups on the backbone of PMAA. Among them, the poly(acrylic acid) (PAA) and its corresponding methyl ester, the poly(methyl acrylate) (PMA), are the most used. Currently, either homo or hetero acrylate block copolymers are also widely employed.

**Figure 1:**
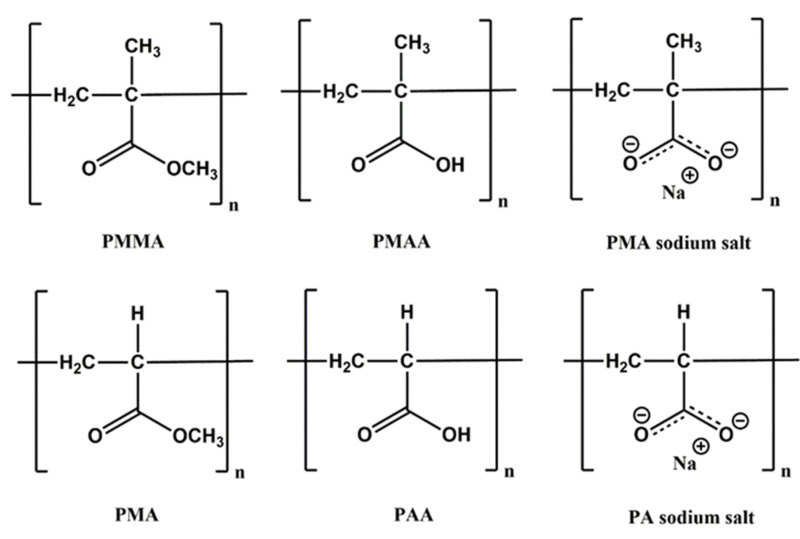
Chemical structures of the poly(acrylic acid) (PAA) and poly(methacrylic acid) (PMAA) and their corresponding sodium salts (PA and PMA sodium salt) and methyl esters (PMA and PMMA).

The chemical structures of acrylic polymers mainly investigated in this review are reported in Figure 1. We will use throughout the manuscript the reported nomenclature, although it could be quite different from that used in the reviewed works. This is done since we are dealing with different chemical structures that sometimes are labeled in the same way.

PMMA is a linear chain polymer that appears macroscopically as an amorphous solid showing a good light transparency and hydrophobic nature, confirmed by the value of the water contact angle of ~68° [39,40]. The hardness or softness of PMMA is determined by the number of methyl groups in the chain, which influences the mobility of the polymer structure. At room temperature PMMA appears as a clear and hard plastic with a glassy aspect, commonly called “Plexiglass”. However, suitable chemical modifications that increase polymer chain mobility led to a white rubber material. PMMA has excellent dimensional stability and can be used in thin-wall applications (tensile modulus: 3171 MPa; tensile elongation: 5%; low mold shrinkage: 0.001–0.004, coefficient of linear thermal expansion: 4.1 × 10^−5^ °F^−1^). Moreover, it has good electrical insulation characteristics such as low dissipation factor (0.04–0.06) and high dielectric strength (100–300 kV/cm). Thus, PMMA can be used for high-voltage electrical insulation up to 20,000 V/mil [41,42].

The good biocompatibility and the easy functionalization strategies allowed a wide employment of PMMA in medical field. It can be fruitfully used as drug/gene nanocarrier or as constituent of medical devices (orthopedic implants, dentures or as reinforced material in ceramics), because it’s non-biodegradable in nature. Furthermore, the loading of antibiotic in PMMA matrix helps to reduce the risk of prosthesis infection, while the optical properties make it a good material for the fabrication of optical devices (implantable ocular or hard contact lenses) [33]. 

PMMA matrix has high extractivity as pertaining to both analytical reagents and analytes in solutions (i.e., solid-phase iodine) without losing its transparency. Thus, highly optically transparent PMMA plates are employed in the spectrophotometric determination of iodides and iodates, the main forms of iodine in food, which deficiency leads to several illnesses [43].

Differently to PMMA, PMA appears as a soft white rubbery material at room temperature, characterized by a lower glass transition temperature (10 °C instead of 105 °C). It is softer than PMMA because its long polymer chains are thinner and smoother and can more easily slide past each other. As a resin, PMA feels leathery upon touch and it is known to have high toughness and good flexibility and adhesion, an elongation of up to 750%, a tensile strength of 6.9 MPa and a refractive index of 1.479. Moreover, PMA shows a higher hydrophobicity than PMMA confirmed by the higher contact angle value of ~73° [39,44]. These peculiar properties together with a good biocompatibility [45] make it ideal for the fabrication of complex plastics with specialized purposes in biomedical, agricultural and electronic applications [46,47,48]. 

Finally, both poly(methacrylic acid) (PMAA) and poly(acrylic acid) (PAA) and their corresponding sodium salts (PMA sodium salt and PA sodium salt) are anionic polyelectrolytes widely employed to prepare three-dimensional structures, called hydrogels, by the addition of small amount of a suitable crosslinking agent. These polymers are classified as “super absorbent polymers” [49], because they can show a water-retention/mass-of-the-polymer ratio up to 500. The total absorbency and swelling capacity of the formed hydrogel depends on both the type and the amount of the crosslinking agent used. The formation of the hydrogel is due to the shape of the polymer obtained and its ability to establish strong interactions with water molecule (i.e., hydrogen bonds) [34,50]. These hydrogels showed bioadhesive, biocompatible and antibacterial properties due to their pendent carboxylic groups. Qi et al. reported a rigid and highly elastic semi-interpenetrating polymer network (semi-IPN) hydrogel made of a poly(acrylamide-co-acrylic acid) copolymer with salecan for oral insulin delivery [51]. Other interesting applications of these polyelectrolytes concern the industry for the waste liquid control, or their use in the medical disposal as diapers sanitary wares. Ultimately, another very promising field of application concerns construction industry where PA sodium salt is used to produce internally cured concrete. When subjected to harsh conditions, such material showed an enhancement of durability and strength performance compared with control samples [52].

The general physico-chemical properties of PAA are reported in Table 1 [53,54,55] in comparison with those already mentioned of PMMA and PMA.

## 3. Polymethacrylate: Applications

### 3.1. Poly(Methyl Methacrylate) (PMMA)

PMMA is an extremely transparent thermoplastic polymer, obtained by polymerization of the monomer methyl methacrylate (MMA). Due to its transparency, aesthetics and resistance to scratches, PMMA can be considered the light alternative to glass. For this reason, it is sometimes referred to as acrylic glass. PMMA is used as an alternative to poly-carbonate (PC) when the previous described features are required and high impact-resistant properties are not essential. These properties make PMMA the polymer of choice in the automotive, lighting, building, cosmetic and medical industries, as we will better describe below. Furthermore, PMMA can be polymerized in the form of microspheres (methacrylic beads) and, depending on the composition, size, glass transition temperature and molecular weight, PMMA beads can be used for various applications: (1) as a matting agent for thermoplastic compounds; (2) as a thickening agent for processes with cast methyl methacrylate and (3) as an additive for covering systems with various decorative effects.

Recently, the mechanical properties of PMMA were reinforced with methylcellulose (MC) [56]. PMMA/MC films can be reused after recovery, maintaining good mechanical properties similar to the original film. These films can be used as sustainable products and possess great potential as an alternative to petroleum-based polymer film products: they are easy to degrade, but with a tensile strength higher than the widely used printing paper, so they could be applied in the packaging material field.

**Figure 2:**
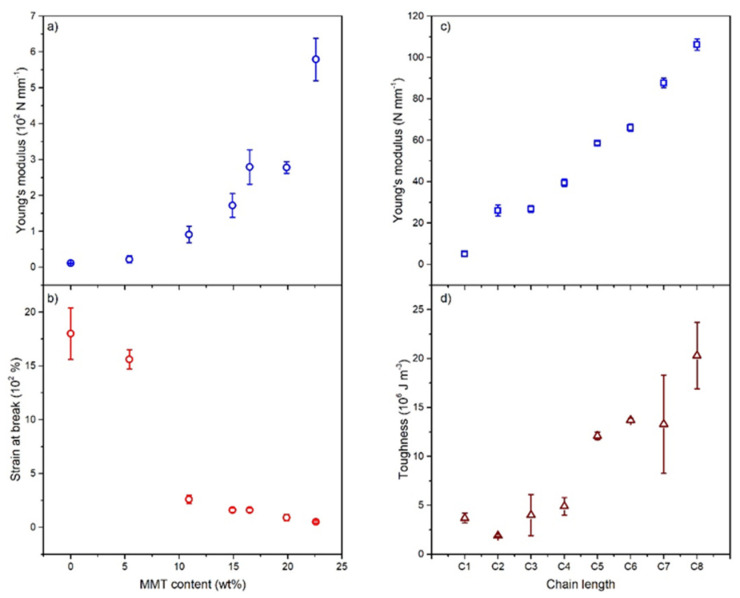
(**a**) Young’s modulus and (**b**) strain at break as a function of MMT content. (**c**) Young’s modulus and (**d**) toughness as a function of the chain length of the grafted PMMA. Adapted from Ref. [57].

PMMA-modified montmorillonite (MMT) nanosheets with grafted polymer chains of variable length were prepared and their mechanical properties (Young’s modulus, tensile strength, toughness and ductility) were evaluated via tensile testing. It emerged that the increase of the overall content of PMMA-grafted MMT resulted in a less ductile material (a decreased strain at break from 18 to 0.5 × 10^2^%), so the Young’s modulus increases of almost two orders of magnitude (from 0.11 to 5.79 × 10^2^ N mm^−1^) as reported in Figure 2a,b. On the other hand, varying the chain length of the grafted PMMA, a significant increase in toughness (from 1.9 to 20.3 × 10^6^ J m^−3^, one order of magnitude with respect to the pristine material) occurs. In this case, the Young’s modulus and the tensile strength depend linearly on the surface-grafted polymer chain length [57] (see Figure 2c,d).

As is well known, the permeability and selectivity characteristics of polymeric membranes are influenced by the molecular structure of the polymer and by the final morphology of the asymmetric membrane, which ranges from finger-like to sponge-like voids. Some efforts are addressed to design innovative membranes for food packaging characterized by improved mechanical properties as well as increased permeability degree without compromising the gas selectivity. Due to its dense nature, PMMA-based membranes and the incorporation of inorganic materials, as additives to the polymer membrane (such as metal oxide nanoparticles), have a high potential for the separation of gas molecules, whose dimensions are of few nanometers [58].

Membrane-based technology is also a promising disinfection method in which microorganisms are retained without any harmful chemical treatment [59]. However, during membrane filtration process, the deposition of microbial cells (biofouling) occurs. PMMA/Ag membranes are effective disinfectants and work for a wide spectrum of bacteria and viruses. The addition of Ag nanoparticles within PMMA matrix induces an increases of membrane permeability, fouling resistance, mechanical and thermal stability, also improving self-cleaning and contaminant degradation properties. Thus, PMMA/Ag nanocomposite film prevents or inhibits growth of microbes, suggesting its use as an antimicrobial or antibacterial agent [60].

**Figure 3:**
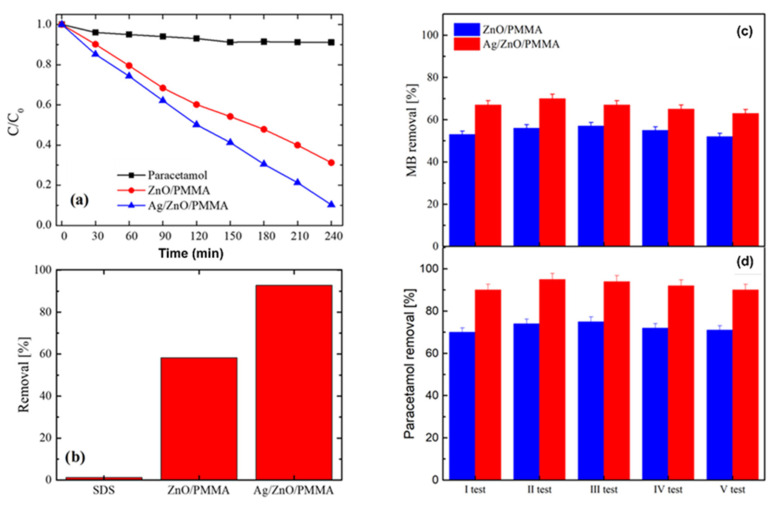
(**a**) Degradation of paracetamol (squares), paracetamol with ZnO/PMMA (circles) and paracetamol with Ag/ZnO/PMMA (triangles) as a function of irradiation time. (**b**) Degradation after 4 h UV radiation exposure for SDS, SDS with ZnO/PMMA and SDS with Ag/ZnO/PMMA. (**c**,**d**) Recyclability of ZnO/PMMA and Ag/ZnO/PMMA after five tests of MB (top) and paracetamol (bottom) photodegradation. Adapted from Ref. [62].

Conventional technologies of water treatment (such as sedimentation, adsorption and filtration) simply transfer the contaminants from one medium to another one, without destroying the pollutants. Unfortunately, some of these methodologies (such as reverse osmosis) have high operating and energetic costs [61]. Heterogeneous photocatalysis is one of the emergent and innovative methods for wastewater treatment. Photocatalysts in the powder form are highly efficient due to their high surface areas, even if nanoparticles can be extremely toxic to aquatic and human life. To overcome this limit, hybrid nanomaterials consisting of PMMA as base material, ZnO layers and Ag nanoparticles have been largely proposed. ZnO has been chosen for its great photocatalytic activity under UV light irradiation, easy growth, low cost and low environmental impact, while Ag nanoparticles were used as an electron scavenger and for its antibacterial properties [62,63,64]. This hybrid nanosystem is able to degrade several organic contaminants such as methylene blue (MB) dye, paracetamol drug and sodium lauryl sulphate (SDS) surfactant, as shown in Figure 3a,b.

Furthermore, it is easy to recycle and reuse (see Figure 3c,d), so that it can be considered suitable for purifying wastewater. This is a great advantage considering that currently one of the major technological obstacles is the recovery of the photocatalytic powders after the wastewater treatment [62].

As remarked in H2020 European Program, concerns about wastewater treatment from nuclear plants are recently increasing. For example, enormous amounts of radioisotopes have been spilled over into the soil and ocean. Hence, it is important to develop a technology for the reduction in nuclear waste along with an automation system to minimize exposure from radioactivity. Clean and quality drinking water is the current demand of undeveloped countries. Available water filters in the local market, like reverse osmosis and micro/nanofilters, are expensive, bulky, inefficient (not potable) and require electricity to operate. Hence, a rapid, potable (filtration rate of about 0.2 L/min is desirable) and cost-effective nanofilter, which does not need electricity to operate, must be engineered to filter the rain and sewerage water from salts, heavy metals and germs, also making it suitable for drinking. Elastic PMMA membrane added with dimethylformamide (DMF) and acetone are successfully synthesized. The resulting electro-spun membrane is tendbal and robust to resist filtration under high stress [65]. Recently, a new web-type electrospun-nanocomposite filter that can effectively adsorb radioactive contaminants was fabricated by Gu et al. [66]. The filter was produced by incorporating Prussian blue (PB) nanoparticles within PMMA nanofibers. PMMA is chosen due to its significant resistance to most environmental parameters. The PB/PMMA nanofiber filter showed the best removal capacity to Cesium (Cs) compared to other radionuclides and the removal efficiency value reached a maximum at pH 7.0. Furthermore, the authors demonstrated the feasibility of filter reuse in the recycling system. This study gives relevant data for future research activities regarding water purification technology through nuclear waste removal.

PMMA surface was recently modified by the atomic layer deposition (ALD) technique. Al_2_O_3_, ZnO and TiO_2_ thin films were deposited by ALD on the polymer surface, first improving and adding new functionalities to polymers and then opening to new opportunities for the surface engineering of high surface-area nanostructures. These PMMA hybrid nanocomposites showed wide versatility for several applications including, energy harvesting, dielectric layers and water purification [67,68].

PMMA is also widely exploited for biomedical applications thanks to its non-toxicity, good biocompatibility, minimal inflammatory reactions when in contact with biological tissues, high mechanical resistance and cheapness [69,70]. To both enhance bioactivity and reduce risk of surgical site infections, PMMA was combined with other materials [71]. Antibiotic-loaded PMMA are largely employed to prevent and treat orthopaedic infections, showing a higher efficiency compared to systemic antibiotic therapies as demonstrated in studies for primary joint replacement surgeries and for severe open fractures [72,73]. However, these delivery systems are poorly efficient to reach a suitable release rate which often decrease to lower dosing levels. The addition of drug-filled cyclodextrin (CD) microparticles into PMMA cement leads to a more efficient release (around 25%), together with a decrease of mechanical strength of PMMA (from 72.4 MPa down to 54.6 MPa). In details, Lu et al. loaded PMMA cement with CD microparticles, previously functionalized with PMMA chains, to reduce the mechanical loss observed with the only addition of unmodified CD microparticles. Moreover, an increase of working time (13.3%) and drug amount refilled (32.1%) in the modified CD loaded PMMA sample compared with PMMA cement were observed [74]. 

**Figure 4:**
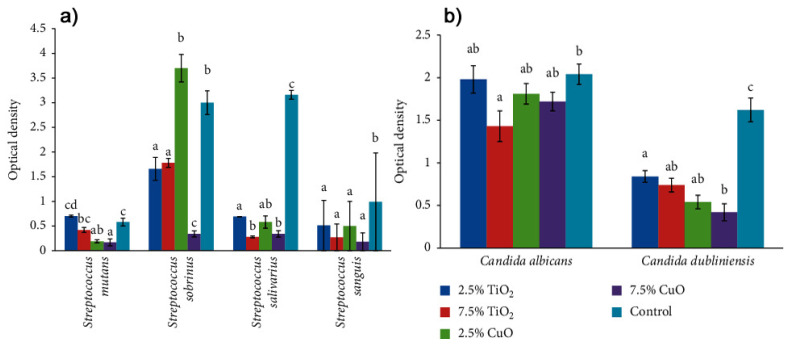
(**a**) Means and standard deviations (*n*  =  5) of optical density of the study groups against different strains of Streptococcus and (**b**) Candida. Vertically within groups, different superscript lowercase letters indicate significant differences between groups in the same column (*p*  ≤  0.05), see Ref. [75] for details. Adapted from Ref. [75].

PMMA is one of the most common materials used for the preparation of removable dentures or cranioplasty implants. Studies revealed that the combination of PMMA with TiO_2_ nanoparticles allows to obtain a hybrid material with improved mechanical and physico-chemical stability together with antibacterial ability due to the presence of metal nanoparticles. The addition of TiO_2_ and CuO in PMMA matrix (at concentrations of 2.5% and 7.5%) led to systems with higher antibacterial activity. In particular, the system with the higher TiO_2_ concentration showed a significantly higher antimicrobial activity compared with the control group assessed against standard strains of yeast [75], as reported in Figure 4.

PMMA hydroxyapatite (PMMA-HA) composite is used to prepare implant materials for cranioplasty thanks to the osteoconductivity effect of HA and the mechanical strength and ease of handling of the PMMA. Shirdar et al. have prepared PMMA modified with HA nanofibers and 2D magnesium phosphate nanosheets, so accelerating bone healing and osseointegration. The authors demonstrated the improving of the cytocompatibility and optimum compressive strength of the nanohybrid composite compared to PMMA [76].

### 3.2. Polymethacrylic Electrolytes (PMAA and PMA Sodium Salt)

Poly(methacrylic acid) (PMAA) is a pH-sensitive polymer. At low pH value ≤ 5.5, the carboxylic acid groups are deprotonated and the polymer is kept in a collapsed state. Conversely, carboxylic acid groups at higher pH value are ionized and the PMAA chains assume an elongated rod-like conformation. The hydrophilicity and biocompatibility of the PMAA and the corresponding sodium salt (PMA sodium salt), together with their ability to absorb water molecules and swell, make these polyelectrolytes interesting materials for biomedical applications. 

Josué A. Torres-Ávalos reported the synthesis of poly(methacrylic acid-co-butyl acrylate) grafted onto functionalized carbon nanotube (CNTs). The authors evidenced that both the presence of CNTs and the change in the inter-associated to self-associated hydrogen bonds balance strongly influence the hydrocortisone release [77].

Best et al. were able to control the mechanical properties of PMA sodium salt hydrogel particles by varying the cross-linker concentration (from 0.1 to 2.0 mg/cc) [78]. In such a way, they tuned the corresponding Young’s modulus between 0.04 and 2.53 MPa and the stiffness of the particles from 1.6 to 28.4 mN m^−1^. They found that the dependence of the concentration of cross-linker added with respect to the quantity of adsorbed polymer follows a Langmuir isotherm. Moreover, we observed that this relationship linearly correlates with the particle mechanical properties. This is considered a pioneering work for the design of innovative soft microparticles particularly useful for biomedical applications. 

**Figure 5:**
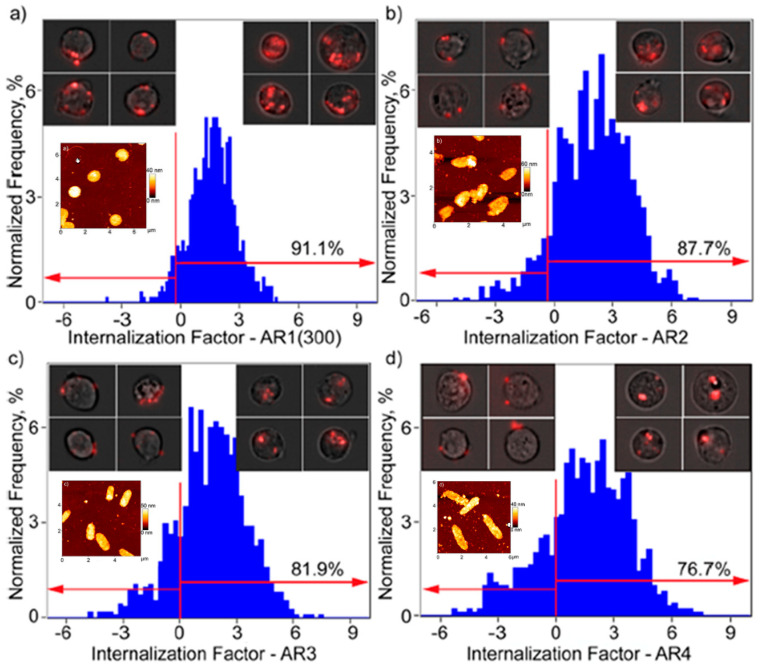
Quantification of the internalization of AF633-labeled PMAA HCs in HeLa cells by imaging flow cytometry. The cells were incubated with capsules at a capsule-to-cell ratio of 100:1 for 6 h at 37 °C, 5% CO_2_. The degree of internalization is expressed as the internalization factor (IF). Each panel shows in the top corners insets an overlay of the bright-field and fluorescence images of cells for two representative areas: capsules bound with the cell membrane (negative IF) and capsules internalized within cells (positive IF). In addition, in each corresponding panel AFM images of PMAA HCs with various aspect ratios are reported. Adapted from Ref. [79].

Shimoni et al. prepared rod-shaped layer-by-layer (LbL)-assembled polymer hydrogel capsules by using silica particles as templates for LbL, with various aspect ratios (ARs) ranging from 1 to 4. By templating spherical and rodlike silica particles, they were able to synthetize disulfide-stabilized PMAA hydrogel capsules (PMAA HCs) [79]. To investigate their behavior at cellular level PMAA HCs were fluorescently labeled by using two reactive dyes, which one is Alexa Fluor 633 C5 maleimide (AF633). A quantitative analysis about the internalization in HeLa cells of AF633-labeled PMAA HCs was performed by using imaging flow cytometry (Figure 5). As shown in Figure 5, an increase in capsule AR induces a decrease of internalization efficiency of the capsules. Then, it can be affirmed that AF633-labeled PMAA HCs with higher ARs content have a higher tendency of binding to the cell surface, rather than being internalized. The present results point out the peculiar role of ARs on cellular processing, paving the way for an innovative use of LbL capsules for biological applications. 

The capability of PMAA to swell in aqueous media was employed to improve the stability of hybrid dental adhesive constituted by Na-MMT nanoclay and silica nanoparticles, where the height density of Na-MMT platelets induces a fast sedimentation process. The polymer chains of the PMAA grafted on the nanoclay surface swell in water environment leading to a separation among the nanoclay platelets, which induces a decrease of the density of the system (being the density of Na-MMT of 2.86 g cc^−1^ and that of the dental bonding system of 1.09 g cc^−1^), to the increase of the dispersion stability (from 1h to several hours). Moreover, the carboxyl groups of PMAA interact with the Ca^2+^ ions of the hydroxyapatite in dentin, so improving the adhesive strength [80]. 

**Figure 6:**
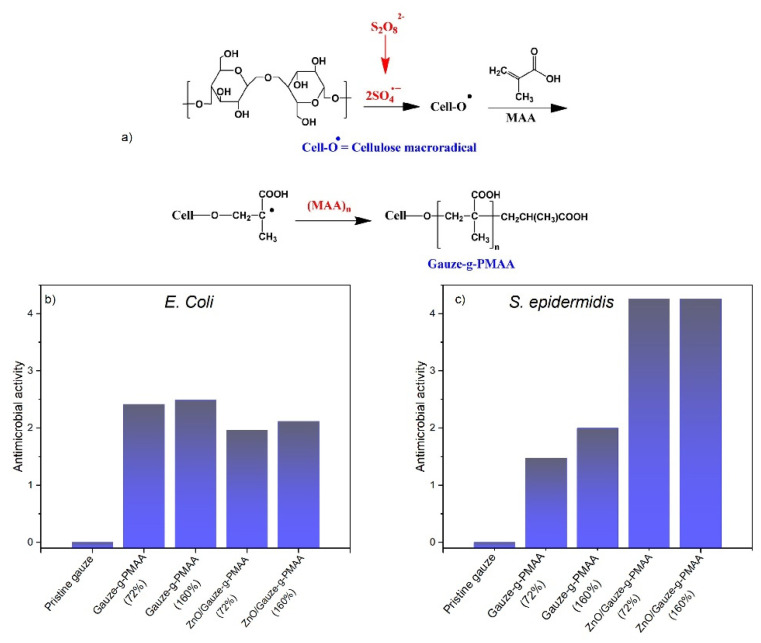
(**a**) Reaction scheme for modification of cotton gauzes with PMAA by free radical polymerization. (**b**,**c**) Antimicrobial activity for pristine and modified cotton gauzes against *E. coli* (**b**) and *S. epidermidis* bacteria (**c**). Reprinted with permission from Ref. [81]. Copyright 2019 Elsevier.

The ability of these carboxylic groups to interact with inorganic nanoparticles or bioactive molecules was also used to prepare carrier systems based on methacrylate polyelectrolytes. Lumbreras-Aguayo and co-workers covalently functionalized cotton gauzes with PMAA to reach medical wound dressings with antimicrobial (see Figure 6) and drug delivery properties [81].

PMAA plays a key role in this medical device because the formation of hydrogen bonds between the carboxyl groups of PMAA and water molecules increases the ability of swell exudates. PMAA cotton gauzes showed superior loading capacity compared with unmodified cotton gauzes (from being negligible up to about 2.5 mg/g for the modified cotton gauze sample with 180% of PMAA), resulting from the formation of hydrogen bonds between carboxylic acid groups of PMAA and the selected drug, along with the swollen state of the grafted PMAA hydrogel, which favors a rapid drug-uptake. The mechanical properties of polymethacrylate hydrogels also endow of a flexibility in the texture structure and a decrease of adhesion formation.

Nowadays, stimuli-responsive controlled-release systems received great attention due to their higher therapeutic efficacy compared to sustained release systems. The possibility to drive the drug release, through either endo/exogenous stimuli at the specific target, reduces the side effect, in turn, enhancing the beneficial for patients [82]. Thanks to the ability to change upon variations in the pH and ionic strength, PMAA is used to prepare pH responsive materials able to protect the bioactive molecules and release them at the target site, improving their bioavailability. Poly(methacrylic acid-co-ethyl acrylate) 1:1 copolymer, called Eudragit L30-D55, is widely employed in pharmaceutical industry thanks to its ability to dissolve above pH 5.5 and keep intact below this pH value [83]. This behavior is due to a change of ionic state as a function of pH variations. A strong electrostatic repulsion occurred among the carboxylic groups leading to the dissociation process at pH > 5.5. On the contrary, at pH < 5.5 the carboxyl groups are protonated (–COOH) and weak electrostatic repulsion between the polymer molecules occurs, so the structure remains intact. Pool et al. [84] employed Eudragit L30-D55 to delivery catechin by the oral route. This biomolecule has several biological activities, but its low water solubility, rapid degradation in the acid environment of stomach, poor absorption by epithelium cells and low overall bioavailability limit its use. Looking to the cumulative release of catechin upon pH variations, the role of the poly(methacrylic acid-co-ethyl acrylate) copolymer as smart carrier is clear. It is able to protect the catechin by the acidic environment of the stomach. Indeed, at low pH (2.5) a slow drug release was detected (about 7%); conversely, the neutral environment of duodenum induces the deprotonation of carboxylic groups with a rapid drug release up to 97% [84].

**Figure 7:**
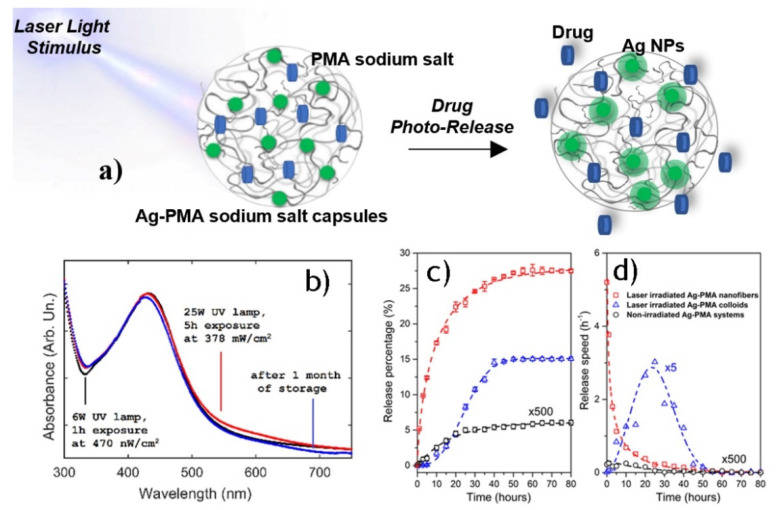
(**a**) Schematic representation of laser light inducing drug release from Ag-PMA sodium salt capsules. (**b**) UV-vis optical absorbance spectra of Ag-PMA sodium salt capsules at different UV irradiation treatments (black and red line) and after one month of storage (blue line) t. (**c**) The release percentage vs time and the corresponding best-fit performed using Weibull function (dashed lines). (**d**) The release speed as the Weibull derivative; some data are multiplied by the indicated factor for clarity. Adapted from Refs. [85,86].

Polymethacrylate electrolytes are also used to prepare light stimuli responsive systems based on noble metal-polymer framework, due to their ability to act as capping agent towards metal clusters. Corsaro et al. developed light responsive drug nanocarriers, based on PMA sodium salt and Ag nanoparticles (see Figure 7a) by a green approach [85,86]. The noble metal nanoparticles were prepared by radiolysis process of water: the reactive species reduce the metal ion (Ag^+^) to zero valent state (Ag), which are stabilized via interactions with carboxylic functional groups of PMA sodium salt and act as capping agent. The small size of the obtained nanoparticles and their high colloidal stability up to three months prove that the polyelectrolyte avoids further nanoparticles aggregation after photoreduction treatment (see Figure 7b).

The drug release process (Figure 7c,d) under irradiation stimulus is allowed by a reorganization of the polymeric matrix due to a hydrogen bonding dissociation process, while the increase of the temperature (up to about 60 °C) induces the aggregation of hydrophobic moieties. This is followed by the expulsion of water molecules by the PMAA network succeeded by the breakdown of polymeric structure [85]. 

Ag-PMA sodium salt nanohybrid system, obtained by a photoreduction process, was also employed by Bonyani et al. to produce an ammonia gas sensor [87]. The good distribution of Ag nanoparticles in the polymer matrix leads to a best electrochemical sensing performance (sensitivity of 130 mA mM^−1^ cm^−2^) with respect to large agglomerates of Ag nanoparticles.

Today, the main negative impact of plastic presence in aquatic environmental is associated with the antibiotic-resistant bacterial strains [88,89,90]. Biofilms are difficult to remove as they have specific defense mechanisms against antimicrobial agents. Hence, antimicrobial surfaces must repel bacteria before they can settle to form a biofilm. An interesting antimicrobial activity is shown by the block copolymers PMMA-b-PMAA against *S. aureus*, *E. coli* and *P. aeruginosa*. This activity only depends on the PMAA content and was not influenced by the copolymer-partner PMMA. In this system, the methacrylic acid groups have a bactericidal effect beside their known adhesion reducing effect [91]. A high amount (about 40 wt.%) of PMAA in the polymer creates a hostile microenvironment for bacteria, causing the death of the microorganisms. The non-leaching acidic surfaces with antimicrobial properties do not need heavy metals or other toxic substances, interesting features for medical, public service and food packaging applications. In addition, a PMAA/montmorillonite (MMT) hydrogel nanocomposite (PMAA/nMMT) acted as an efficient sorbent for sequestration of pharmaceutical contaminants. Specifically, economic feasibility, better sorption capacity (152.65 for AMX and 152.86 mg/g for DF) and efficient regeneration and reusability even after four consecutive sorption−desorption cycles make PMAA/nMMT as a potential sorbent for amoxicillin and diclofenac uptake from the aqueous phase [92]. 

**Figure 8:**
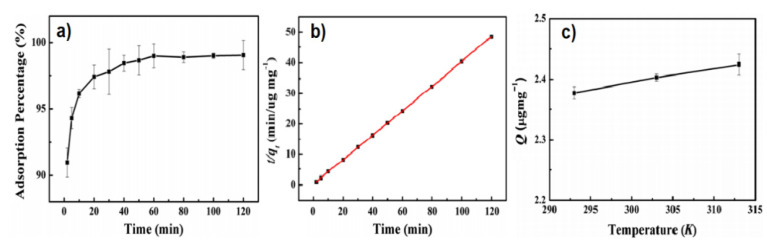
(**a**) Adsorption percentage of BCG onto poly(GMA-MAA-EDMA)-CHT vs time; (**b**) Pseudo-second-order kinetic model plots for the adsorption data reported in (**a**); (**c**) Effect of temperature on the adsorption of BCG onto poly(GMA-MAA-EDMA)-CHT. (All the shown trends refer to the following conditions: dosage of adsorbent: 10 mg, concentration of BCG: 5 μg cc^−1^, adsorption time: 40 min, pH 2.0). Adapted from Ref. [93].

Liu et al. have prepared chitosan poly(methacrylate) composites, containing methacrylic acid fragments, to adsorb bromocresol green (BCG) from aqueous solutions [93]. Bromocresol is used as a pH indicator and as a tracking dye for DNA agarose gel electrophoresis. It is stable and not easily degradable for its complex aromatic molecular structure, therefore it has an impact on the aquatic life and food web even in low concentrations. The authors have found that the adsorption percentage of bromocresol green with chitosan poly(methacrylate) composites remained above 97% after three times of recycling test (see Figure 8). The best performance was observed at pH 2; at this value a decrease of positive charge of BCG is expected, favouring electrostatic attraction and absorption of BCG onto the chitosan poly(methacrylate) surface. At pH > 2.0 a decrease of absorption efficiency was observed due to an electrostatic repulsion between BCG and anionic surface of absorbent composite [93]. 

## 4. Polyacrylate: Applications 

### 4.1. Poly(Methyl Acrylate) (PMA) 

Acrylate polymers such as PMA, bearing hydrophilic ester pendent groups on the hydrophobic backbone, can easily form stable monolayers at the water surface due to their amphiphilic nature. PMA monolayers show expanded-type isotherm (i.e., the surface pressure detected at a large surface area which slowly increases with decreasing of the area per repeated unit occupied on the surface). Otherwise, PMMA isotherms are the condensed-type ones, featured as the fastest increment in the surface pressure. Comparing PMA with PMMA structure, it emerges that the α-methyl group (α-CH_3_) exerts a significant impact on the poly(acrylates) interfacial behaviour [94]. These differences affect the structure–property relationships of the polymer monolayers at a liquid interface and, in turn, their performance in some technological applications, as described below.

**Figure 9:**
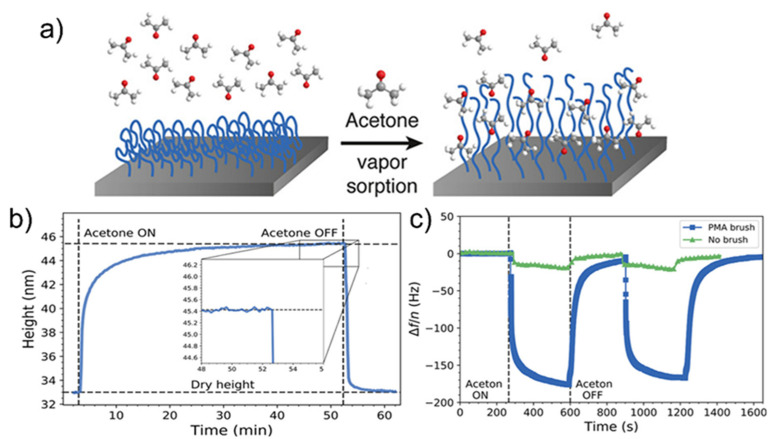
(**a**) Schematic picture of acetone sensing by brush PMA. (**b**) Swelling response of the dense brush after exposure to acetone vapor at t = 0 and to dry nitrogen gas at t = 53 min and (**c**) the measured change in oscillation frequency (fundamental) upon exposure to acetone vapor for a PMA brush versus a crystal with a brush as measured by quartz crystal microbalance. Adapted from Ref. [95].

The importance to reduce the food waste is essential to fight the increasing request of food and the huge amount of resources consumption that such problem entails. With this aim, the development of new, compatible sensors to monitor the food quality and its degradation plays a pivotal role. In such sense, one of the markers correlated to the starting of food degradation is acetone. By observing the good solubility of PMA in acetone, Horst and coworkers developed a brush PMA-based sensor for the detection of acetone in gas [95] (see Figure 9a). The solvation of PMA is probably due to dipole interactions between the ester functional group of the polymer and carbonyl group of acetone molecules. An increase of the weight to 33% was found, which is comparable to the one reported for PMMA (30%) in a previous work [96]. Considering that PMMA and PMA have the same ester moiety, the obtained result confirmed the supposed solvation process. The response of the brush PMA to acetone vapor occurs in few seconds and reached the equilibrium within 40 min (see Figure 9b,c), differently from spin coated-polymer films which required from several hours to days [97]. The authors also demonstrated that the swelling ratio is independent by the polymer length and grafting density, which is important for the large-scale applications. 

The low glass transition temperature (T_g_) of PMA, correlated to the good chain mobility, makes it an interesting material as polymer membrane in quasi-solid electrolyte for Dye-Sensitized Solar Cells (DSSC). It was demonstrated that, for amorphous polymer electrolytes, the ionic conduction is confined above their T_g_. This highlights the pivotal role of the chain mobility in the conductivity mechanism: by enhancing the chain mobility the ionic conductivity is improved. Gelled PMA showed a light to electricity conversion efficiency of 7.17%, significantly higher compared to other polymers with higher T_g_ values. In this way, Fathy et al. prepared PMA nanofibers membrane to form quasi-solid state DSSCs. Thanks to the easy crossing of liquid electrolyte through the pores of membranes, a high ionic conductivity of 2.4 × 10^−3^ S cm^−1^ at room temperature and a solar-electricity conversion efficiency of 1.4% at an illumination intensity of 100 mW cm^−2^ were evaluated. The electrolyte solution was well encapsulated within the membrane structure with a better long-term stability compared to standard liquid electrolytes [98].

PMA is also a well known cold flow improver (CFI) used to overcome the problem about the block of the fuel pipe and filter of the diesel engine when biodiesels are employed. Biodiesels contain saturated fatty acid methyl esters which easily crystalized or gelled at low temperatures due to their high-melting point [99]. Adding very small amount of PMA (0.04%) to biodiesel is an efficient and economic strategy to lower the cold filter plugging point of oils [100]. This tiny quantity of added PMA reduced the biodiesel pour point and cold filter plugging point of 8 and 6 °C, respectively. Essentially, PMA influences the crystallization process, avoiding the formation of crystals larger than few microns at temperatures of −10 and −20 °C [101]. We outline the best performance of PMA in comparison with other CFI agents reported in literature [102,103]. 

To guarantee the uniform dissolution of PMA in biodiesel, considering its high viscosity, Xu et al. mixed PMA with Span80 dispersant agent at different mass ratios (1:0, 4:1, 2:1, 1:1, 1:2, 1:4 and 0:1). The best result was reached at 2:1 mass ratio with a decrease of 7 °C cold filter plugging point compared to biodiesel with pure PMA (4 °C) [104].

Disinfectant medical nanocapsules are prepared by using PMA. Tanpantree et al. demonstrate that the average size, shell thickness and encapsulation efficiency of the chlorhexidine digluconate (CHD)-PMA nanocapsules were significantly improved when PMA, with an average molecular weight (M_w_) of 550 k, was used as shell material. The encapsulation efficiency of the CHD-PMA nanocapsules, dispersed in 0.5% *w*/*v* sodium dodecyl sulfate aqueous solution, was greater than 90%.

The need to control and reproduce in vitro models of Fibronectin (FN) networks including new synthetic materials able to serve as bio-inspired scaffolds for tissue engineering, has driven the efforts for the identification of cell-free routes able to induce FN fibrillogenesis. Adsorption of individual FN molecules onto specific surface induces exposure of self-assembly sites to lead FN fibril assembly process. FN showed an interesting different distribution on PMA and poly(ethyl acrylate) (PEA), that differ only by one methyl group in the side chain, giving substrates with similar physico-chemical properties. FN forms globular aggregates on PMA surface; conversely, an interconnected network on PEA was observed. The higher coverage of FN on PEA surface was confirmed by contact angle measurements. Indeed, a higher contact angle hysteresis of FN on PEA surface due to an estimated stronger decrease of the receding angles compared to FN on PMA, (about 90° compared to 65° on PMA, for concentrations greater than 5 mg/cc). In the latter case, FN keeps a globular conformation. These data outlined the potentiality of alkyl acrylate polymers as chemical surfaces to generate FN fibrils where the increasing formation of a FN network on PEA led to higher levels of myogenesis; instead, a gradient of globular FN molecules on PMA did not alter cell differentiation. Thus, PMA was efficiently used as a control polymer [105,106].

**Figure 10:**
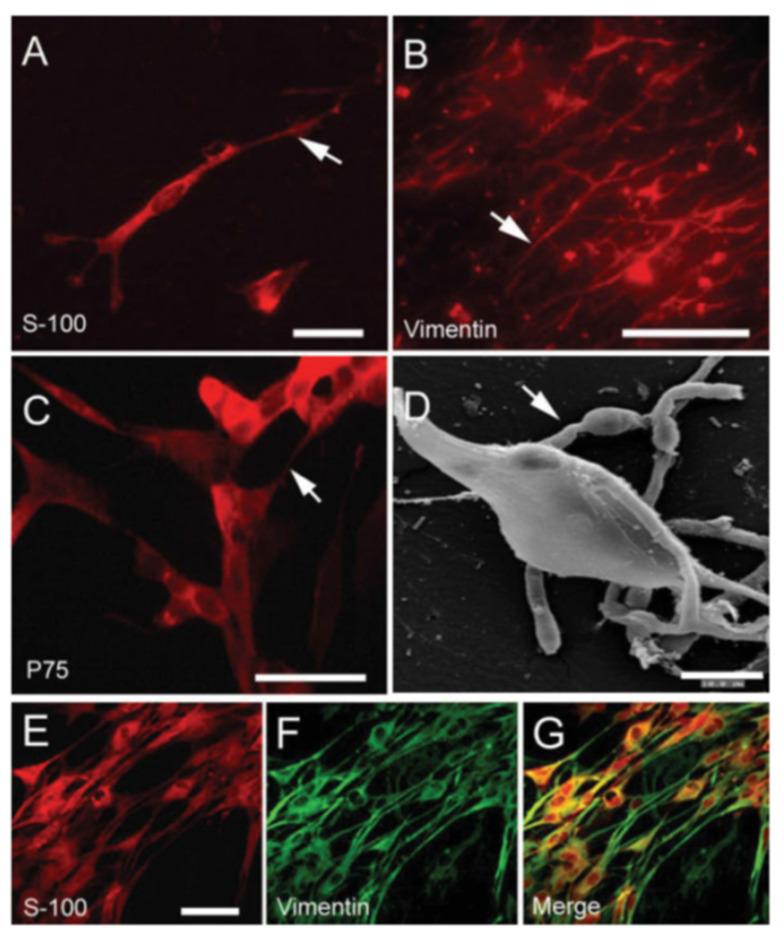
SCs cultured onto a P(EA-co-HEA) 90/10 substrate. Immunocytochemistry assays for the markers S100 (**A**), vimentin (**B**) and p75 (**C**); the arrow highlights a suitable cell viability of glial cells with cytoplasm extensions onto the synthetic substrate. (**D**) Scanning electron micrograph of cultured SCs with a usual spindled and shaped morphology and with cytoplasm extensions (white arrows). Double inmmunocytochemistry for S100 (**E**) and vimentin (**F**) show that almost all cultured cells (>95%) expressed both markers (**G**). Scale bar: (**A**): 50 μm, (**B**): 100 μm, (**C**): 60 μm, (**D**): 10 μm, (**E**–**G**): 50 μm. Reprinted with permission from Ref. [107]. Copyright 2007 Wiley Periodicals, Inc.

PMA and PEA polymers were also suitable substrates to promote cell attachment and proliferation of Schwann cells (SCs) in vitro compared to other more hydrophilic polymers. Soria et al. observed an inverse correlation between cellular colonization with wettability [107]. This could be explained considering that the adsorption process is driven both by several kinds of non-covalent interactions, which take place between the extracellular matrix (ECM) proteins and the polymer surfaces and by entropy, involving the release of bound water molecules from the proteins while they unfold to be adsorbed on the polymer surface. Certainly, the second mechanism favours materials with hydrophobic character, whereas the interplay of both mechanisms influences the amount of the adsorbed proteins and their conformation. All these characteristics explain the better performance of both PMA and PEA compared to hydrophilic polymers like poly(hydroxyethyl acrylate) (PHEA). So, favourable substrates able to promote SC attachment, maintaining their morphological characteristics [107], are provided (see Figure 10). Therefore, PMA and PEA could be used for the fabrication of more complex scaffolding structures for peripheral nervous system repair and tissue engineering applications.

**Figure 11:**
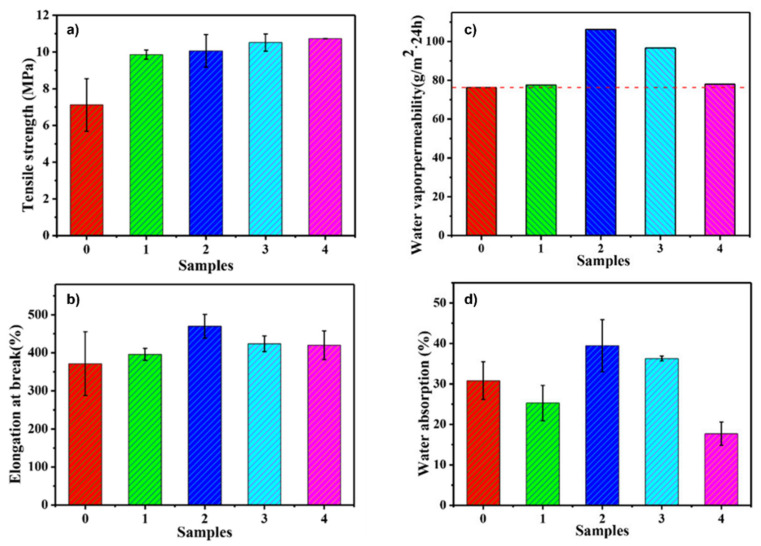
Effect of fillers on mechanical properties (**a**) Tensile strength, (**b**) Elongation at break, and on (**c**) water vapor permeability and (**d**) water resistance of composite films (0. PMA, 1. PMA/SiO_2_, 2. PMA/KH550-SiO_2_, 3. PMA/KH560-SiO_2_ and 4. PMA/KH570-SiO_2_). Adapted from Ref. [108].

Wu et al. [108] have investigated the effect of the surface structure of SiO_2_ particles, treated with polysiloxanes with similar chain length (KH550, KH560 or KH570), on the mechanical characteristic and water vapor permeability of a poly(methyl acrylate)/SiO_2_ nanocomposites. PMA/KH570-SiO_2_ and PMA/KH550-SiO_2_ showed the highest tensile strength (Figure 11a) and elongation at break (Figure 11b) at the same filler content. Instead, KH550-SiO_2_ spheres significantly improve water vapor permeability (Figure 11c) of polyacrylate film. This is explained taking into account the different hydrophilic and hydrophobic surface of SiO_2_ and mainly since the different functional groups on the SiO_2_ surface leads to different interfacial interactions with PMA, from which the different mechanical properties. Finally, the water absorption of PMA/KH550-SiO_2_ and PMA/KH560-SiO_2_ (on the contrary of PMA/KH570-SiO_2_, composite films) is higher than that of PMA (Figure 11d).

Rahman et al. [109] proposed PMA-grafted and poly(acrylonitrile)-grafted cellulose for wastewater treatments. The pure cellulosic materials were extracted from waste fibers and then modified by free radical grafting reaction. The obtained materials show up to 98% and 90%, respectively, removal of copper and other metal ions, which are promising results mainly for industrial wastewater.

Polymer-based food packaging is composed by a multi-layer system where a layer acts as a barrier against gas diffusion. The low-density polyethylene (LDPE) is one of the most used technical commodity polymers since it provides excellent barrier properties against moisture as well as low barrier characteristics against oxygen and flavour; characteristics necessary to keep the product fresh ensuring the consumers safe. Nevertheless, some efforts are made to engineer new innovative multi-layer composites. Linear PMA has been grafted to montmorillonite (MMT) nanosheets via a surface-confined grafting-through *Reversible Addition-Fragmentation chain Transfer* (RAFT) polymerization. The topology of the grafted PMA has been altered from a linear to a mixture of star-shaped and linear PMA. In some cases, PMA has been cross-linked through the addition of hydrogen bonding sites within a small proportion of the monomer units. Specifically, three different coatings were applied and compared: nanosheets grafted with linear PMA (MMT-PMA_L_), a mixture of star and linear PMA (MMT-PMA_S_) and with linear PMA that contained 2.6 mol% of carboxyethyl acrylate, a hydrogen bonding comonomer (MMT-PMA_HB_). The authors found that the gas permeability can be controlled through the coating’s thickness (see Table 2) and that the polymer-grafted MMT nanosheets, cast to the surface of Low-density polyethylene (LDPE), form a μm-thin coating which effectively decreases gas diffusion [110]. 

### 4.2. Poly(Acrylic) Electrolytes (PAA and PA Sodium Salt)

Poly(acrylic) electrolytes are largely applied thanks to their specific chemical features. For example, the differences observed between poly(acrylic acid) (PAA) and poly(methacrylic acid) (PMAA) are correlated to a transition from a rather compact coil to an extended one, occurring during ionization in the case of PMAA. All these differences are ascribed to the presence of hydrophobic methyl side groups in the PMAA chain. However, there is still some unresolved question regarding the real nature of forces responsible for the compact PMAA structure at low ionization values. Really, a balance between cohesion forces, due to hydrophobic groups and repulsive coulombic interactions occurs. The conductivity of the PMA polyelectrolytes depends on the amount and the type of ions, in turn, affected by several variables such as polymer polarity, water content, salt/ions and hydrogel structure [111]. 

**Figure 12:**
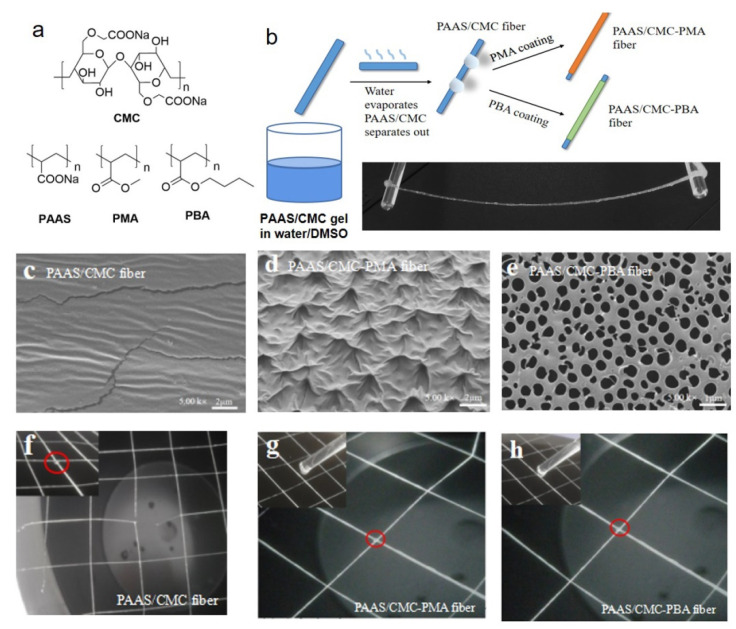
(**a**) Chemical structure of carboxymethyl cellulose (CMC), sodium polyacrylate (PAAS), PMA and polybutyl acrylate (PBA). (**b**) Preparation of conductive hydrogel fibers by gel spinning. Inserted image shows the photo picture of as-prepared PAAS/CMC fiber. SEM images of the surface of (**c**) PAAS/CMC fiber, (**d**) PAAS/CMC-PMA fiber and (**e**) PAAS/CMC-PBA fiber. The water resistance test of hydrogel fibers: (**f**) PAAS/CMC fiber web is quickly dissolved by liquid water, water droplets would not damage the hydrophobic PAAS/CMC-PMA fiber (**g**) and PAAS/CMC-PBA fiber (**h**). Red circles represent thorn-like structure indicating cratering effects after encountering liquid water. Adapted from Ref. [114]. Note that PAAS in this Figure corresponds to our PA sodium salt.

A growing interest is currently focused on the development of stretchable and soft conductive materials for electronic devices by modification of polyacrylic electrolyte. Conductive ionic hydrogels, which are easy to synthetize, cheap and biocompatible, have attracted much attention [112]; however, their low robustness limits their applications [113]. To overcome this issue, Wang et al. designed a gel fiber composed by PA sodium salt, which guarantees the electrically conductive and stretchability and sodium carboxymethyl cellulose (CMC), which contribute to improve fibers robustness [114]. The physical crosslinking structure between the soft polyelectrolyte (PA sodium salt, 4% weight) and the rigid polyelectrolyte (CMC, 1% weight), dissolved into water/DMSO mixture (water: DMSO 78:22 by weight), give rise to the hydrogel fibers. The last ones are covered with PMA, which acts as insulating material, protecting them by the breakup when they are immersed in liquid water, but also contributing to improve their strength (see Figure 12). PMA-covered hydrogel fibers show an ionic conductivity of ∼0.35 S/m slightly lower than that of uncovered fibers (∼0.55 S/m) and the electrical conductivity is kept also after stretch tests, demonstrating their potentiality as stretchable-conductive based fibers in electronic devices.

To replace commonly liquid electrolytes, great interest is focused on the design of solid polymer electrolyte (SPE) thanks to their ease of manufacturing, wide operation temperature range, low volatility, high energy density and so on [115,116]. In this field, PAA is an interesting material because it has high charge density due to the carboxylic groups, high stability both in acid and basic media and strong adhesive properties [117]. Certainly, the ionization degree depends on the pH value: in acid media, the PAA has a low charge density due to the low dissociation degree; conversely, at pH > 5 the carboxylic groups are deprotonated, resulting in high polymer ionization, which allows to establish hydrogen bonds, electrostatic interactions and coordination with metal nanoparticles. Moreover, it showed high ionic conductivity and high electrochemical stability [118]. 

Abu Saied et al. grafted PAA onto plasma-activated poly(vinyl chloride) (PVC) membrane surfaces, reaching a membrane with a strong hydrophilic surface due to the carboxyl groups of PAA. The authors proved that the thermal decomposition of the PAA-PVC membrane does not start below 250 °C [119]. As expected, after the addition of PAA a considerable increase of ion exchange capacity was detected (from about 0.03 up to 0.12 mmol/g) together with an improvement of the polar solvent uptake (from about 6 up to 25% for water). Hosseini et al. [120] fabricated polyacrylic acid copolymer polymethyl methacrylate/PVC (PAA-co-PMMA/PVC) based heterogeneous cation exchange membranes using graft polymerization technique. Increasing the ratio of emulsifier to monomers improved the ion exchange capacity and the electrochemical properties of these composite membranes. Moreover, in recent studies on thermoplastic ion exchange membranes, the effects of incorporating magnetic nanoparticles (CoFe_2_O_4_) and bis(8-hydroxyquinoline)zinc (ZnQ_2_) nanoparticles in PVC matrix on membranes morphology, swelling, transport number and permselectivity were reported [121].

Khan et al. developed polymer hydrogel based on PAA and LiClO_4_ salt at four different molar concentrations (0.5 M, 1 M, 1.5 M and 2 M) [122]. The carboxylate anion groups of PAA interact with the Li^+^ ions forming a double complex network. Moreover, the ability of carboxylic groups to establish hydrogen bonds with water molecules provides flexible and stretchable hydrogel electrolyte. The authors investigated the electrochemical performance of the fabricated cells (activated carbon/PAA-LiClO_4_/activated carbon) and found the best results when the LiClO_4_ concentration is 1.5 M. In particular, a maximum specific capacitance of 115 F g^−1^ at 3 mV s^−1^ and 132.20 F g^−1^ at 50 mA g^−1^, with an energy and power density of 18.36 Wh kg^−1^ and 1000 W kg^−1^, were respectively evaluated. These results point out the potentiality of PAA-LiClO_4_ hydrogel electrolytes as a competitive system for application in supercapacitors. 

**Figure 13:**
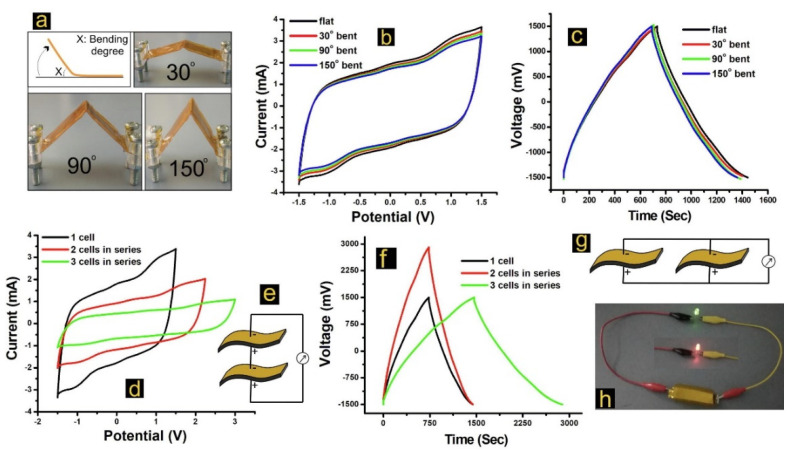
(**a**) Picture of flexible supercapacitor with different bending angles, (**b**) The CV of bent device with PAA-Co7 in different angles, (**c**) galvanostatic charge/discharge (GCD) profiles under different bending angles, (**d**,**e**) CV voltammograms of cells (1–3 cells) connected in series at a rate of 10 mV s^−1^, (**f**,**g**) comparison of GCD diagrams of cells with series and parallel connections, (**h**) picture of red-blue-green Light Emitting Diode (RBG LED) connected to charged supercapacitor. Reprinted with permission from Ref. [123]. Copyright 2021 Elsevier.

The intercalation of redox active units in electrolytes enhances the capacitance of the capacitor device. Cevik and co-workers prepared a low-cost supercapacitor by a redox mediated PAA matrix [123]. The authors intercalated different amount of cobalt (Co) into PAA-based electrolyte (3, 5, 7 and 10 correspond to the doping fraction (*w*/*w*) of Co in PAA) and the so obtained redox activated polychelates (PAA-Co) are stabilized by the ionic interaction between Co^2+^ and the carboxyl groups of the polymer. All samples are characterized by good flexibility at four different bending states (flat, 30°, 90° and 150°, see Figure 11a), thermal stability with no weight change up to 100 °C and an optimum high ionic conductivity of 3.15 × 10^−4^ S cm^−1^. The electrolytes were used to prepare flexible supercapacitors, which showed significant capacitance retention of 90% up to 10.000 cycles and good cycle robustness up on bending at various angles (see Figure 13b–f). The best electrolyte, which contains a 7% table (*w*/*w*) of Co, showed a capacitance of 341.33 F g^−1^, an energy density of 21.25 Wh kg^−1^ at a power density of 117.69 W kg^−1^. So, it was used to prepare a supercapacitor which successfully operated the red-blue-green (RGB) LED light (Figure 13h). 

For the first time, Al Munsur and co-workers [124] prepared Nafion-based proton-exchange membranes based on crosslinked PAA and PVA. PAA is selected due to its water selectivity and permeability, while the crosslinking process is needed to avoid the water dissolution of the PAA-based membranes. The authors worked at two different mass ratios (PAA-PVA-Nafion, 2.5:2.5:95 or 5:5:90) and found that the best performances are reached at the 2.5:2.5:95 PAA-PVA-Nafion mass ratio. This membrane is characterized by a cylindrical morphology, leading to an improvement of thermal, mechanical and dimensional stability of the membrane. This system showed higher bound water content (90.7% of total water content) due to its good ability to retain water molecules compared to recast Nafion membrane (76.6%). Thus, the PAA-PVA crosslinked membrane showed significantly improved conductivity (about 180 mS/cm at 80 °C) and cell performance (maximum power density of about 1.2 W cm^−2^) compared to recast Nafion membrane (maximum power density of about 0.9 W cm^−2^). These data pointing out the potentiality of PAA-PVA-Nafion system for proton-exchange membrane fuel cell and proton-exchange membrane water electrolyzer (PEMWE) applications, overcoming the limits of Nafion, like proton conductivity and oxidative stability, which are not yet satisfactory [124]. 

**Figure 14:**
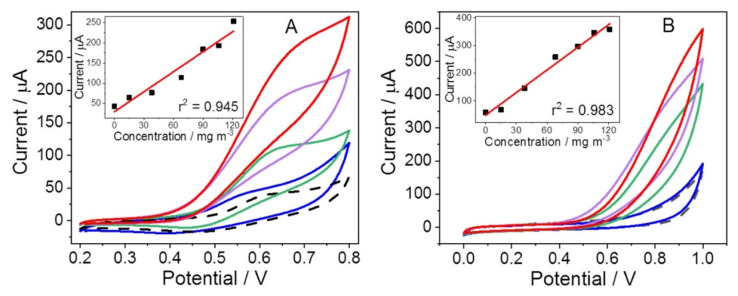
Cyclic voltammograms obtained at the MnO_2_/PAA (**A**) and PAA (**B**) sensors for successive increments of gaseous H_2_O_2_ concentrations in the range of 15–121 mg m^−3^ (voltammograms for 15, 68, 106 and 121 mg m^−3^ are shown) together with a background response (dashed line). The insets show the corresponding calibration plots. Reprinted with permission from Ref. [125]. Copyright 2021 Elsevier.

Isailovíc et al. developed two electrochemical sensors based on PAA for measuring gaseous H_2_O_2_, a key molecule in several field from food production to biomedicine, including cosmetic and pharmaceutical industry [125]. One sensor is constituted by PAA sensing membrane deposited onto the commercial supporting screen-printed electrodes and the other one involves only a PAA sensing membrane (we outline that the other one’s working electrode is electrochemically pre-modified with MnO_2_). A good sensitivity in the low mg m^−3^ concentration range was evaluated with a detection limit of 3 μg m^−3^ and 2 μg m^−3^ for the sensors with and without MnO_2_, respectively. Both systems showed good selectivity towards H_2_O_2_ interfering gaseous compounds and a superior electroanalytical performance of the PAA sensor (see Figure 14), with the advantage of an easier preparation route. This sensitive and selective gas sensing system could be employed for several applications including environmental monitoring, clinical diagnostics, reduction of occupational health hazards and so on. 

Thanks to its water-solubility, biocompatibility and interesting mechanical properties, PAA is also used to improve the tensile strength of several systems for bone tissue engineering. Cheng et al. have introduced PAA in PVA hydrogel to overcome the poor tensile strength and tissue adhesion typical of pure PVA hydrogels [126]. The cold-drawn technique employed by authors allows it to reach a PVA/PAA hydrogel strong interconnected by a high number of hydrogen bonds, resulting in excellent tensile strength close to one of natural cartilage (~17 MPa, [127]). The addition of PAA improves the interfacial adhesion between the gel and tissue and influences the roughness of hydrogel surface leading to a biocompatible system with potential application as cartilage tissue substitute.

PMA is also used to improve the mechanical properties of calcium phosphate cement. To optimize the properties of bone cement for medical application, Thaitalay et al. studied the cement solution at different *v*/*v*% ratio of Na_2_HPO_4_ and PAA [128]. The best ratio was found to be 30:70 *v*/*v*% (PAA: Na_2_HPO_4_), improving the compressive strength and reaching a high cell viability, while the phase transformation is kept for bioactivity giving a suitable composite as bone substitution material. 

Jiang et al. outline the key role of PMA to regulate biomineralization events. PAA can behave as non-collagenous proteins due to the high amount of carboxylic acid groups, regulating the hydroxyapatite (HA) formation [129]. They investigated the effect PAA on amorphous mediated HA crystallization process and observed an opposite trend at low (20–50 mg/cc) and high (60–90 mg/cc) PAA concentration. At the low concentration, PAA is mainly incorporated into amorphous calcium phosphate (ACP) and smaller ACP nanoparticles, which mediated the crystallization process. This gives several sites for HAP nucleation on ACP surface, promoting the crystallization process. Conversely, high amount of PAA covers the nucleation sites on ACP surface, changing the morphology of ACP and blocking the HA crystallization process. The ability of PAA to influence HA crystallization could be an interesting tool to regulate the development and the properties of bone substitutes [130].

**Figure 15:**
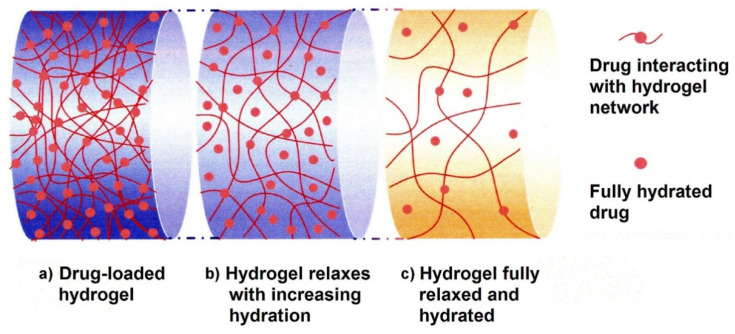
Schematic illustration of the process of drug release from hydrogel. Reprinted with permission from Ref. [131]. Copyright 2017 Elsevier.

Analogously to PMAA, PAA is used to prepare control drug delivery systems pH responsive. A hydrogel constituted by chitosan crosslinked with PAA and loaded with amoxicillin and meloxicam was prepared by Wang and co-workers. For this system, the drug release amount increases with pH (from 1.2 to 7.4) [131]. At lower pH values, the carboxylic acid groups of the PAA are almost protonated, establishing hydrogen bonds among them and, in turn, leading to a compact structure where the diffusion of water molecules is hindered. Otherwise, at pH 7.4, the carboxylic groups are deprotonated and their negative charges induce electrostatic repulsion between the PAA acid chains. Thus, the diffusion of water molecules inside the hydrogel is favoured stabilizing it. This phenomenon increases the swelling of hydrogel promoting the drug release process that can be explained by three main steps, as shown in Figure 15. The first step corresponds to the drug-loaded hydrogel containing a minimum amount of water. At this stage, the hydrogel exhibits the lowest flexibility, pores are small and drug mobility is modest (Figure 15a). In the second step, hydrogel relaxes due to increasing water hydration and becomes more flexible. Consequently, pores become larger and drug mobility enhances (Figure 15b). In the last step, the hydrogel is totally relaxed being completely hydrated; pores have their maximum size and the rate of drug diffusion from the hydrogel reaches the highest value (see Figure 15c).

Soliman et al. developed a cancer diagnostic imaging device based on polyethylene oxide–PAA nanogel, which is radio-labeled with the imaging radioisotope ^99^mTc (metastable nuclear isomer of technetium-99) and then conjugated with folic acid, which act as targeting agent. Biodistribution data indicated a high selective uptake of the prepared complex in cancer muscle respect to the healthy one for both intratumor and intravenous injections, highlighting its potentiality as nanotheranostic device [132].

## 5. Discussion

In the last decade, the efforts of the scientific community are focused to prepare synthetic mimic nature and compatible polymeric based products to ensure the ecosystem survival. The majority of global plastics consists of synthetic polymer with carbon–carbon backbones whose low cost led to their massive use in virtually all commercial and industrial fields. However, their environmental persistence has resulted in a massive reservoir of plastic waste in the environment. The currently marketed bio-degradable plastics cannot still entirely replace fossil fuel-based plastics, even if they seem to be the only possibility to ensure the assimilation of the carbon-based degraded product as a food source for soil or aquatic microorganisms. Since the current state of the biodegradable plastics in the zero-waste-focused circular economy contest is not totally clear, great attention is focused on the recycling of traditional polymers, even if some characteristics of the polymers based on polymethyl acrylate unit really limit their efficiently and eco-friendly applicability in future. As described in the previous sections, PMMA has a low impact strength (thus, some impact modifiers must be used to improve this property) it has poor resistance to attack by ketones, esters, ethers, aromatic and halogenated solvents. All interesting properties make PMMA suitable for various applications requiring high hardness, even if limit its “destruction or conversion” at the end of the product’s life. In addition, PMMA costs more per pound than its competition and commodity thermoplastics such as polystyrene. This accounts for the lowest usage volume of PMMA (about 1.5 billion pounds per years) compared to 20 billion pounds per year for polystyrene. For all these reasons, PMMA is not easily recyclable and it is classified as a group 7 thermoplastic among recycled plastics [133].

No universal approach to energy-efficient thermoset recycling is adopted nowadays, even if many industrial realities are moving in this direction. For instance, as a result of the demand for sustainable packaging by end consumers, the industry is paying close attention to innovations that will allow for the recycling of materials used in the manufacturing of said packaging. An attractive circular polymer economy approach is to transform polymers back into monomers and purify them for repolymerization (namely, chemical recycling to monomer (CRM)). This is considered the most suitable approach in terms of the simplification of recycling procedures, energy cost and the preparation of sustainable polymeric materials with a broad range of physico-chemical properties [134]. For example, recyclable catalyst-free polymethacrylate networks containing dynamic dialkylamino disulphide linkages (namely BiTEMPS) are synthesized by Bin Rusayyis et al. [135]. The authors demonstrated that the reprocessable polymers with dynamic covalent bonds (both on step-growth polymer networks or based on full cross-link density recovery of reprocessable networks prepared from only monomers via addition polymerization) are characterized by thermoplastic-like properties. 

**Figure 16:**
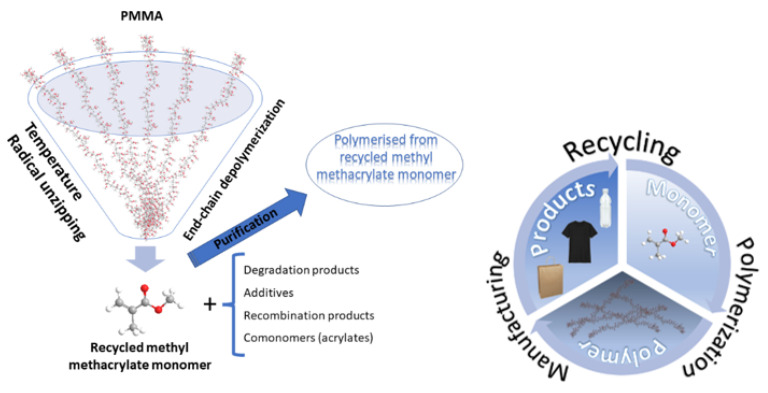
PMMA depolymerisation chemistry.

In the last years, an EU-funded P2L project (https://cordis.europa.eu/project/id/856103, 10 December 2021) found a solution by recycling PMMA into its monomer unit MMA which can be reused, thus creating a circular economy for PMMA (see Figure 16). The technology is also affordable and environmentally friendly; therefore, it supports a profitable and sustainable PMMA economy [136,137].

This is a good approach to avoid the generation of any carbon-centered radicals during reprocessing which could lead to deleterious termination reactions and thus loss of cross-link density with reprocessing. Generally, from a recycling perspective, these systems should focus on the development of dissociative exchange mechanisms as they can allow a viscosity drop that is sufficient for (thermoplastic) reprocessing and separation of the matrix in polymer thermoset composite products [138]. 

Among the polymers available, PMAA is indicated as one of those viable for producing C-S-H/polymer complexes. For example, the addition of PMAA to calcium silicate hydrate (C-S-H) resulted in changes in structural packing and, in turn, an increase of the nanocomposite micro-nanomechanical properties. This is important for the production of materials that are more efficient in relation to the binding forces, thus improving the tensile strength, also avoiding cracking problems (i.e., for concrete and mortar widely used by the construction industry and/or teeth and bones reconstruction in biomedical applications). 

To minimize the waste disposal problems, another proposed approach is to modify existing synthetic polymers by blending with a wide range of biopolymers. Among them, cellulose derivatives can be used being thermoplastics in nature, easy to process and thus can be blended with synthetic polymers. Further, cellulose derivatives are readily biodegradable by microorganisms that utilize cellulose enzymes, even if cellulose requires the presence of esterases due to the additional acetyl groups. Thereby, biodegradation could be induced in the synthetic polymer [139].

Beside that, the post-functionalization seems to be a good strategy to preserve the integrity of the polymer skeleton and to reach a new route for upcycling of adhesive and coating waste into value-added products. A good functionalization of sterically differentiated acrylate copolymers and polymeric chain ends was carried out by Hou et al. [140] by the 1,5,7-triazabicyclo[4.4.0]undec-5-ene (TBD)-catalyzed or metal-catalyzed transesterification of PMA and PMMA, combined with a reversible-addition fragmentation chain transfer. On the other hand, the controlled de-functionalization may also favour some polymers modifications: the elimination of pendant ester group in the PMA structure gives a linear hydrocarbon polymer with methyl pendants, which corresponds to polypropylene (PP). This reaction is performed by employing B(C_6_F_5_)_3_-catalyzed deoxygenation in presence of silane [141]. In addition, functionalized adsorbents could be obtained by grafting of acrylic acid, poly(propylene imine) dendrimer and acrylonitrile onto PP.

Specifically, the recycling of methyl acrylic polyelectrolytes could be based on a different principle. It is an issue still opened which must be treated together a greater understanding of the mechanism of polyelectrolyte complexation. Thermodynamic studies indicate that the entropy, due to release of counterions, is the key driving force for strong polyelectrolyte complexation. Moreover, entropic contributions dominate the free energy of complex formation for strong polyelectrolytes but are less important than energetic contributions when weak electrostatic coupling or weak polyelectrolytes occur. Specifically, the free energy of polyelectrolyte complex formation is driven by polymer association, which should also arise in systems with large charge spacings or bulky counterions, both of which act to weaken ion–polymer binding. Hence, a more complete understanding of these mechanisms could be fundamental to design appropriate polyelectrolytes recycling methodologies [142].

Temperature-responsive polymer brushes are applied for production of selective membranes, smart bioactive surfaces, separation systems, mechanical actuators and transducers [143]. For example, cholesterol-based polymers that have capabilities to create different self-assemblies in a broad temperature range and solvent regimes are used. Thermal and mechanical properties of poly(methyl methacrylate) are improved using mono-acrylated isosorbide as a bio-based monomer [144]. On the other hand, competitive (meth)acrylates and the corresponding polymers made from bio-renewable resources with a specific focus on lignocellulose are adopted [145]. Further, Stetsyshyn et al. [146] synthesized grafted liquid crystalline polymer brushes with cholesterol side chains poly(cholesteryl methacrylate), PChMa), which shows smooth temperature tuning of surface anchoring for a nematic liquid crystal (ZLI-4119, from EM Industries Inc., Hawthorne, NY 10532, USA) and, thus, useful for future liquid crystalline display production.

Although there are margins to introduce innovative, recyclable and recycled products based on methacrylate or acrylate polymers for the food industry and biomedical field, major challenges are facing in being cost-effective in the production of nontoxic nano-delivery systems, effective formulations that are safe for human consumption and drug treatments. Selectivity in this case remains a challenge that researchers have to solve, for example, through specific functionalization of the polymeric matrix. The effective implementation of recycling processing methods and their blending effects depend directly on the understanding of acrylic polymers behavior, blending techniques and properties. In this respect, challenges result from their sensitivity to varying factors during production, such as temperature-dependent effects and properties such as mechanical and chemical. Moreover, we should take into account that methacrylate- or acrylate-based blends are often dependent on the morphology, rheology and properties of the material blended. Hence, we considered it essential to analyze in depth the fundamental complexation and then recycling mechanisms, taking into account the multiple time-scale transient processes that take place under the different experimental conditions which, ultimately, determine the new polyacrylates properties.

## 6. Conclusions

In this review we thoroughly investigated the chemical structure, physical properties of methacrylate and acrylate polymers and their recent applications in several fields were discussed. We outlined the advantages of using these polymers and the suitable strategies to overcome their drawbacks with the aim to guide into their demand and future prospect. Thus, we hope that this review could be a support for the research to engineering innovative value-added products based on this class of polymers. This must be done considering the current state of the art and identifying the fundamental mechanisms to be still clarified for a decisive turning point to improve human and ecosystem life.

## Figures and Tables

**Table 1:** Overview about the physico-chemical properties of PMMA, PMA and PAA.

	PMMA	PMA	PAA
Glass transition temperature	105 °C	10 °C	~103 °C
Contact angle	~68°	~73°	~22°
Refractive Index	1.491	1.479	1.527
Tensile strength	3171 MPa	6.9 MPa	~0.5 MPa
Tensile elongation	5%	750%	~340%

**Table 2:** Average durations ∆t_400→600mbar_—the time difference between 400 and 600 mbar in the sample chamber—for LDPE-foils coated with MMT nanosheets modified with PMA_L_, PMA_S_ or PMA_HB_. Errors are obtained as the standard deviation of at least three measurements. Reprinted from Ref. [110].

Sample	Number of Coating Cycles	∆t_400→600mbar_/min
LDPE	0	38 ± 11
LDPE + MMT-PMA_L_	1	79 ± 11
LDPE + MMT-PMA_S_	1	74 ± 16
LDPE + MMT-PMA_HB_	1	77 ± 8
LDPE + MMT-PMA_L_	3	86 ± 4
LDPE + MMT-PMA_S_	3	96 ± 8
LDPE + MMT-PMA_HB_	3	104 ± 8

## Data Availability

No new data were created or analyzed in this study. Data sharing is not applicable to this manuscript.

## References

[1] Reshmy R., Philip E., Madhavan A., Sindhu R., Pugazhendhi A., Binod P., Sirohi R., Awasthi M.K., Tarafdar A., Pandey A. (2021). Advanced biomaterials for sustainable applications in the food industry: Updates and challenges. Environ. Pollut..

[2] Soltani Firouz M., Mohi-Alden K., Omid M. (2021). A critical review on intelligent and active packaging in the food industry: Research and development. Food Res. Int..

[3] Lee J., Seol W., Anoop G., Samanta S., Unithrattil S., Ahn D., Kim W., Jung G., Jo J. (2021). Stabilization of Ferroelectric Phase in Highly Oriented Quinuclidinium Perrhenate (HQReO_4_) Thin Films. Materials.

[4] Fazio E., Spadaro S., Corsaro C., Neri G., Leonardi S.G., Neri F., Lavanya N., Sekar C., Donato N., Neri G. (2021). Metal-Oxide Based Nanomaterials: Synthesis, Characterization and Their Applications in Electrical and Electrochemical Sensors. Sensors.

[5] Sylvain R., Vendier L., Bijani C., Santoro A., Puntoriero F., Campagna S., Sutra P., Igau A. (2013). Evidence of the unprecedented conversion of intermolecular proton to water bridging of two phosphoryl ruthenium complexes. New J. Chem..

[6] Santoro A., Holub J., Fik-Jaskółka M.A., Vantomme G., Lehn J.-M. (2020). Dynamic Helicates Self-Assembly from Homo- and Heterotopic Dynamic Covalent Ligand Strands. Chem.—A Eur. J..

[7] Mancuso A., Barattucci A., Bonaccorsi P., Giannetto A., La Ganga G., Musarra-Pizzo M., Salerno T.M.G., Santoro A., Sciortino M.T., Puntoriero F. (2018). Carbohydrates and Charges on Oligo(phenylenethynylenes): Towards the Design of Cancer Bullets. Chem.—A Eur. J..

[8] Caccamo D., Currò M., Ientile R., Verderio E.A., Scala A., Mazzaglia A., Pennisi R., Musarra-Pizzo M., Zagami R., Neri G. (2020). Intracellular Fate and Impact on Gene Expression of Doxorubicin/Cyclodextrin-Graphene Nanomaterials at Sub-Toxic Concentration. Int. J. Mol. Sci..

[9] Jeong G.H., Sasikala S.P., Yun T., Lee G.Y., Lee W.J., Kim S.O. (2020). Nanoscale Assembly of 2D Materials for Energy and Environmental Applications. Adv. Mater..

[10] Foti C., Mineo P.G., Nicosia A., Scala A., Neri G., Piperno A. (2020). Recent Advances of Graphene-Based Strategies for Arsenic Remediation. Front. Chem..

[11] Liang W., Wang B., Cheng J., Xiao D., Xie Z., Zhao J. (2021). 3D, eco-friendly metal-organic frameworks@carbon nanotube aerogels composite materials for removal of pesticides in water. J. Hazard. Mater..

[12] Lugger S.J.D., Houben S.J.A., Foelen Y., Debije M.G., Schenning A.P.H.J., Mulder D.J. (2021). Hydrogen-Bonded Supramolecular Liquid Crystal Polymers: Smart Materials with Stimuli-Responsive, Self-Healing, and Recyclable Properties. Chem. Rev..

[13] La Mantia F.P., Ceraulo M., Testa P., Morreale M. (2021). Biodegradable Polymers for the Production of Nets for Agricultural Product Packaging. Materials.

[14] Liu G.-Y., Sun W.-F., Lei Q.-Q. (2021). Charge Injection and Dielectric Characteristics of Polyethylene Terephthalate Based on Semiconductor Electrodes. Materials.

[15] Celentano W., Neri G., Distante F., Li M., Messa P., Chirizzi C., Chaabane L., De Campo F., Metrangolo P., Baldelli Bombelli F. (2020). Design of fluorinated hyperbranched polyether copolymers for ^19^F MRI nanotheranostics. Polym. Chem..

[16] Marć M., Bystrzanowska M., Pokajewicz K., Tobiszewski M. (2021). Multivariate Assessment of Procedures for Molecularly Imprinted Polymer Synthesis for Pesticides Determination in Environmental and Agricultural Samples. Materials.

[17] Cruz Sanchez F.A., Boudaoud H., Hoppe S., Camargo M. (2017). Polymer recycling in an open-source additive manufacturing context: Mechanical issues. Addit. Manuf..

[18] Karak N. (2009). Fundamentals of Polymers: Raw Materials To Finish Products.

[19] Cywar R.M., Rorrer N.A., Hoyt C.B., Beckham G.T., Chen E.Y.X. (2021). Bio-based polymers with performance-advantaged properties. Nat. Rev. Mater..

[20] Moshood T.D., Nawanir G., Mahmud F., Mohamad F., Ahmad M.H., Abdul Ghani A. (2021). Expanding Policy for Biodegradable Plastic Products and Market Dynamics of Bio-Based Plastics: Challenges and Opportunities. Sustainability.

[21] Vera M., Mella C., Urbano B.F. (2020). Smart Polymer Nanocomposites: Recent advances and Perspectives. J. Chil. Chem. Soc..

[22] Aguilar M.R., San Román J., Aguilar M.R., San Román J. (2019). Chapter 1—Introduction to Smart Polymers and Their Applications. Smart Polymers and Their Applications.

[23] Wieszczycka K., Staszak K., Woźniak-Budych M.J., Litowczenko J., Maciejewska B.M., Jurga S. (2021). Surface functionalization—The way for advanced applications of smart materials. Coord. Chem. Rev..

[24] Reddy M.S.B., Ponnamma D., Choudhary R., Sadasivuni K.K. (2021). A Comparative Review of Natural and Synthetic Biopolymer Composite Scaffolds. Polymers.

[25] Stuart M.C., de Vries R., Lyklema H., Lyklema J. (2005). Chapter 2—Polyelectrolytes. Fundamentals of Interface and Colloid Science.

[26] Khan N., Brettmann B. (2019). Intermolecular Interactions in Polyelectrolyte and Surfactant Complexes in Solution. Polymers.

[27] Petrila L.-M., Bucatariu F., Mihai M., Teodosiu C. (2021). Polyelectrolyte Multilayers: An Overview on Fabrication, Properties, and Biomedical and Environmental Applications. Materials.

[28] Gupta A.K., Sahu D.K., Giri S. (2020). Study of Electrical Conductivity of Poly(Methyl Acrylate) PMA Polymer Thin Film. Int. J. Adv. Res. Sci. Technol. (IJARST).

[29] Eygi M.S., Ateşok G. (2008). An investigation on utilization of poly-electrolytes as dispersant for kaolin slurry and its slip casting properties. Ceram. Int..

[30] Gretz M., Plank J. (2010). Hybrid additives for construction applications, fabricated through layer-by-layer adsorption of polycondensate type superplasticizers on latex templates. Colloids Surf. A Physicochem. Eng. Asp..

[31] Virga E., de Grooth J., Žvab K., de Vos W.M. (2019). Stable Polyelectrolyte Multilayer-Based Hollow Fiber Nanofiltration Membranes for Produced Water Treatment. ACS Appl. Polym. Mater..

[32] Hartmann H., Krastev R. (2017). Biofunctionalization of surfaces using polyelectrolyte multilayers. BioNanoMaterials.

[33] Ajekwene K. (2020). Properties and Applications of Acrylates.

[34] Mahon R., Balogun Y., Oluyemi G., Njuguna J. (2019). Swelling performance of sodium polyacrylate and poly(acrylamide-co-acrylic acid) potassium salt. SN Appl. Sci..

[35] Amina S.J., Guo B. (2020). A Review on the Synthesis and Functionalization of Gold Nanoparticles as a Drug Delivery Vehicle. Int. J. Nanomed..

[36] Ouni O.A., Subbiahdoss G., Scheberl A., Reimhult E. (2021). DNA Polyelectrolyte Multilayer Coatings Are Antifouling and Promote Mammalian Cell Adhesion. Materials.

[37] Xing H., Wang X., Xiao S., Zhang G., Li M., Wang P., Shi Q., Qiao P., Liu H. (2017). Osseointegration of layer-by-layer polyelectrolyte multilayers loaded with IGF1 and coated on titanium implant under osteoporotic condition. Int. J. Nanomed..

[38] Holmes P.F., Bohrer M., Kohn J. (2008). Exploration of polymethacrylate structure–property correlations: Advances towards combinatorial and high-throughput methods for biomaterials discovery. Prog. Polym. Sci..

[39] Shi Z., Zhang Y., Liu M., Hanaor D.A.H., Gan Y. (2018). Dynamic contact angle hysteresis in liquid bridges. Colloids Surf. A Physicochem. Eng. Asp..

[40] Zhang C., Zhou Y., Shao T., Xie Q., Xu J., Yang W. (2014). Hydrophobic treatment on polymethylmethacrylate surface by nanosecond-pulse DBDs in CF_4_ at atmospheric pressure. Appl. Surf. Sci..

[41] Zhang H.Q., Jin Y., Qiu Y. (2015). The optical and electrical characteristics of PMMA film prepared by spin coating method. IOP Conf. Ser. Mater. Sci. Eng..

[42] Abu Hassan Shaari H., Ramli M.M., Mohtar M.N., Abdul Rahman N., Ahmad A. (2021). Synthesis and Conductivity Studies of Poly(Methyl Methacrylate) (PMMA) by Co-Polymerization and Blending with Polyaniline (PANi). Polymers.

[43] Gavrilenko N.A., Fedan D.A., Saranchina N.V., Gavrilenko M.A. (2019). Solid phase colorimetric determination of iodine in food grade salt using polymethacrylate matrix. Food Chem..

[44] Zhang Y., Fan H., Wang Y., Zuo B., Zhang W., Wang S., Wang X. (2015). Influence of the linkage type between the polymer backbone and side groups on the surface segregation of methyl groups during film formation. Soft Matter.

[45] Yahata C., Suzuki J., Mochizuki A. (2019). Biocompatibility and adhesive strength properties of poly(methyl acrylate-co-acrylic acid) as a function of acrylic acid content. J. Bioact. Compat. Polym..

[46] Halacheva S.S., Adlam D.J., Hendow E.K., Freemont T.J., Hoyland J., Saunders B.R. (2014). Injectable Biocompatible and Biodegradable pH-Responsive Hollow Particle Gels Containing Poly(acrylic acid): The Effect of Copolymer Composition on Gel Properties. Biomacromolecules.

[47] Junior C.R.F., de Moura M.R., Aouada F.A. (2017). Synthesis and Characterization of Intercalated Nanocomposites Based on Poly(methacrylic acid) Hydrogel and Nanoclay Cloisite-Na^+^ for Possible Application in Agriculture. J. Nanosci. Nanotechnol..

[48] Feng Y., Smith C.S., Burkett S.L. (2017). Process for patterning features in poly(acrylic acid) for microelectronic applications. J. Micromech. Microeng..

[49] Sultana S., Sumon K.I., Noor H.P., Ajmotgir W.M., Sarker K.U., Hasan R. (2017). Sswelling and Physico-Mechanical Properties of Synthesized Sodium Polyacrylate Hydrogels. Int. J. Adv. Res..

[50] Arens L., Barther D., Landsgesell J., Holm C., Wilhelm M. (2019). Poly(sodium acrylate) hydrogels: Synthesis of various network architectures, local molecular dynamics, salt partitioning, desalination and simulation. Soft Matter.

[51] Qi X., Yuan Y., Zhang J., Bulte J.W.M., Dong W. (2018). Oral Administration of Salecan-Based Hydrogels for Controlled Insulin Delivery. J. Agric. Food Chem..

[52] Manzur T., Iffat S., Noor M.A. (2015). Efficiency of Sodium Polyacrylate to Improve Durability of Concrete under Adverse Curing Condition. Adv. Mater. Sci. Eng..

[53] Abate A.R., Krummel A.T., Lee D., Marquez M., Holtze C., Weitz D.A. (2008). Photoreactive coating for high-contrast spatial patterning of microfluidic device wettability. Lab Chip.

[54] Dehbari N., Tavakoli J., Singh Khatrao S., Tang Y. (2017). In situ polymerized hyperbranched polymer reinforced poly(acrylic acid) hydrogels. Mater. Chem. Front..

[55] Eisenberg A., Yokoyama T., Sambalido E. (1969). Dehydration kinetics and glass transition of poly(acrylic acid). J. Polym. Sci. Part A-1: Polym. Chem..

[56] Wang Y., Duo T., Xu X., Xiao Z., Xu A., Liu R., Jiang C., Lu J. (2020). Eco-Friendly High-Performance Poly(methyl methacrylate) Film Reinforced with Methylcellulose. ACS Omega.

[57] Rauschendorfer J.E., Thien K.M., Denz M., Köster S., Ehlers F., Vana P. (2021). Tuning the Mechanical Properties of Poly(Methyl Acrylate) via Surface-Functionalized Montmorillonite Nanosheets. Macromol. Mater. Eng..

[58] Kianfar E., Cao V. (2021). Polymeric Membranes on base of PolyMethyl Methacrylate for Air Separation: A review. J. Mater. Res. Technol..

[59] Nguyen T., Roddick F.A., Fan L. (2012). Biofouling of Water Treatment Membranes: A Review of the Underlying Causes, Monitoring Techniques and Control Measures. Membranes.

[60] Awad M.A.G., Hendi A.A., Khalid O., Ortashi M., Soliman D.W.A. (2018). Synthesis of Silver—PMMA Nanocomposite Film using Herbal Extract. U.S. Patent.

[61] Pearson J.L., Michael P.R., Ghaffour N., Missimer T.M. (2021). Economics and Energy Consumption of Brackish Water Reverse Osmosis Desalination: Innovations and Impacts of Feedwater Quality. Membranes.

[62] Di Mauro A., Farrugia C., Abela S., Refalo P., Grech M., Falqui L., Nicotra G., Sfuncia G., Mio A., Buccheri M.A. (2020). Ag/ZnO/PMMA Nanocomposites for Efficient Water Reuse. ACS Appl. Bio Mater..

[63] Di Mauro A., Farrugia C., Abela S., Refalo P., Grech M., Falqui L., Privitera V., Impellizzeri G. (2020). Synthesis of ZnO/PMMA nanocomposite by low-temperature atomic layer deposition for possible photocatalysis applications. Mater. Sci. Semicond. Process..

[64] Shu Z., Zhang Y., Yang Q., Yang H. (2017). Halloysite Nanotubes Supported Ag and ZnO Nanoparticles with Synergistically Enhanced Antibacterial Activity. Nanoscale Res. Lett..

[65] Ahmad B., Hamza A., Ahmed S., Najam Z., Ishtiaq A. (2019). Synthesis and Characterization of PMMA Nanofibers for Filtration of Drinking Water. J. Mech. Contin. Math. Sci..

[66] Gu G.E., Bae J., Park H.S., Hong J.-Y. (2021). Development of the Functionalized Nanocomposite Materials for Adsorption/Decontamination of Radioactive Pollutants. Materials.

[67] Forte M.A., Silva R.M., Tavares C.J., Silva R.F.E. (2021). Is Poly(methyl methacrylate) (PMMA) a Suitable Substrate for ALD? A Review. Polymers.

[68] Yin J., Deng B. (2015). Polymer-matrix nanocomposite membranes for water treatment. J. Membr. Sci..

[69] Ali U., Karim K.J.B.A., Buang N.A. (2015). A Review of the Properties and Applications of Poly(Methyl Methacrylate) (PMMA). Polym. Rev..

[70] Kim S.J., Choi B., Kim K.S., Bae W.J., Hong S.H., Lee J.Y., Hwang T.-K., Kim S.W. (2015). The Potential Role of Polymethyl Methacrylate as a New Packaging Material for the Implantable Medical Device in the Bladder. BioMed Res. Int..

[71] Marin E., Boschetto F., Zanocco M., Honma T., Zhu W., Pezzotti G. (2021). Explorative study on the antibacterial effects of 3D-printed PMMA/nitrides composites. Mater. Des..

[72] Ayre W.N., Birchall J.C., Evans S.L., Denyer S.P. (2016). A novel liposomal drug delivery system for PMMA bone cements. J. Biomed. Mater. Res. Part B Appl. Biomater..

[73] Seligson D., Berling S. (2015). Antibiotic-laden PMMA bead chains for the prevention of infection in compound fractures: Current state of the art. Eur. J. Orthop. Surg. Traumatol..

[74] Lu C.-Y., Church D.C., Learn G.D., Pokorski J.K., von Recum H.A. (2021). Modified Cyclodextrin Microparticles to Improve PMMA Drug Delivery Without Mechanical Loss. Macromol. Biosci..

[75] Giti R., Zomorodian K., Firouzmandi M., Zareshahrabadi Z., Rahmannasab S. (2021). Antimicrobial Activity of Thermocycled Polymethyl Methacrylate Resin Reinforced with Titanium Dioxide and Copper Oxide Nanoparticles. Int. J. Dent..

[76] Shirdar M.R., Taheri M.M., Qi M.-L., Gohari S., Farajpour N., Narayanan S., Foroozan T., Sharifi-Asl S., Shahbazian-Yassar R., Shokuhfar T. (2021). Optimization of the Mechanical Properties and the Cytocompatibility for the PMMA Nanocomposites Reinforced with the Hydroxyapatite Nanofibers and the Magnesium Phosphate Nanosheets. Materials.

[77] Torres-Ávalos J.A., Cajero-Zul L.R., Vázquez-Lepe M., López-Dellamary F.A., Martínez-Richa A., Barrera-Rivera K.A., López-Serrano F., Nuño-Donlucas S.M. (2021). Synthesis of Poly(methacrylic acid-co-butyl acrylate) Grafted onto Functionalized Carbon Nanotube Nanocomposites for Drug Delivery. Polymers.

[78] Best J.P., Cui J., Müllner M., Caruso F. (2013). Tuning the Mechanical Properties of Nanoporous Hydrogel Particles via Polymer Cross-Linking. Langmuir.

[79] Shimoni O., Yan Y., Wang Y., Caruso F. (2013). Shape-Dependent Cellular Processing of Polyelectrolyte Capsules. ACS Nano.

[80] Solhi L., Atai M., Nodehi A., Imani M. (2020). Poly(Methacrylic Acid) Modified Spherical and Platelet Hybrid Nanoparticles as Reinforcing Fillers for Dentin Bonding Systems: Synthesis and Properties. J. Mech. Behav. Biomed. Mater..

[81] Lumbreras-Aguayo A., Meléndez-Ortiz H.I., Puente-Urbina B., Alvarado-Canché C., Ledezma A., Romero-García J., Betancourt-Galindo R. (2019). Poly(methacrylic acid)-modified medical cotton gauzes with antimicrobial and drug delivery properties for their use as wound dressings. Carbohydr. Polym..

[82] Corsaro C., Mallamace D., Neri G., Fazio E. (2021). Hydrophilicity and hydrophobicity: Key aspects for biomedical and technological purposes. Phys. A Stat. Mech. Its Appl..

[83] Kshirsagar S., Bhalekar M., Umap R. (2009). In Vitro In Vivo Comparison of Two pH Sensitive Eudragit Polymers for Colon Specific Drug Delivery. J. Pharm. Sci. Res..

[84] Pool H., Luna-Barcenas G., McClements D.J., Mendoza S. (2017). Development of Polymethacrylate Nanospheres as Targeted Delivery Systems for Catechin within the Gastrointestinal Tract. J. Nanopart. Res..

[85] Corsaro C., Neri G., Mezzasalma A.M., Fazio E. (2021). Weibull Modeling of Controlled Drug Release from Ag-PMA Nanosystems. Polymers.

[86] Neri G., Corsaro C., Fazio E. (2020). Plasmon-Enhanced Controlled Drug Release from Ag-PMA Capsules. Molecules.

[87] Bonyani M., Mirzaei A., Leonardi S.G., Neri G. (2016). Silver Nanoparticles/Polymethacrylic acid (AgNPs/PMA) Hybrid Nanocomposites-Modified Electrodes for the Electrochemical Detection of Nitrate Ions. Measurement.

[88] Schreiber C., Zacharias N., Essert S.M., Wasser F., Müller H., Sib E., Precht T., Parcina M., Bierbaum G., Schmithausen R.M. (2021). Clinically Relevant Antibiotic-Resistant Bacteria in Aquatic environments—An Optimized Culture-based Approach. Sci. Total Environ..

[89] Sharma V.K., Johnson N., Cizmas L., McDonald T.J., Kim H. (2016). A Review of the Influence of Treatment Strategies on Antibiotic Resistant Bacteria and Antibiotic Resistance Genes. Chemosphere.

[90] Zhou C.-s., Wu J.-w., Dong L.-l., Liu B.-f., Xing D.-f., Yang S.-s., Wu X.-k., Wang Q., Fan J.-n., Feng L.-p. (2020). Removal of Antibiotic Resistant Bacteria and Antibiotic Resistance Genes in Wastewater Effluent by UV-activated Persulfate. J. Hazard. Mater..

[91] Gottenbos B., Grijpma D.W., van der Mei H.C., Feijen J., Busscher H.J. (2001). Antimicrobial effects of positively charged surfaces on adhering Gram-positive and Gram-negative bacteria. J. Antimicrob. Chemother..

[92] Khan S.A., Siddiqui M.F., Khan T.A. (2020). Synthesis of Poly(methacrylic acid)/Montmorillonite Hydrogel Nanocomposite for Efficient Adsorption of Amoxicillin and Diclofenac from Aqueous Environment: Kinetic, Isotherm, Reusability, and Thermodynamic Investigations. ACS Omega.

[93] Liu D., Yuan J., Li J., Zhang G. (2019). Preparation of Chitosan Poly(methacrylate) Composites for Adsorption of Bromocresol Green. ACS Omega.

[94] Hong Y., Zhou H., Qian W., Zuo B., Wang X. (2017). Impact of the α-Methyl Group (α-CH_3_) on the Aggregation States and Interfacial Isotherms of Poly(acrylates) Monolayers at the Water Surface. J. Phys. Chem. C.

[95] Horst R.J., Brió Pérez M., Cohen R., Cirelli M., Dueñas Robles P.S., Elshof M.G., Andreski A., Hempenius M.A., Benes N.E., Damen C. (2020). Swelling of Poly(methyl acrylate) Brushes in Acetone Vapor. Langmuir.

[96] Orski S.V., Sheridan R.J., Chan E.P., Beers K.L. (2015). Utilizing vapor swelling of surface-initiated polymer brushes to develop quantitative measurements of brush thermodynamics and grafting density. Polymer.

[97] Koziara B.T., Akkilic N., Nijmeijer K., Benes N.E. (2016). The Effects of Water on the Morphology and the Swelling Behavior of Sulfonated Poly(ether ether ketone) Films. J. Mater. Sci..

[98] Fathy M., Kashyout A.B., El Nady J., Ebrahim S., Soliman M.B. (2016). Electrospun Polymethylacrylate Nanofibers Membranes for Quasi-solid-state Dye Sensitized Solar Cells. Alex. Eng. J..

[99] Mei D., Tan W., Yuan Y. (2013). Crystal Precipitation Law of Biodiesel based on Thermodynamic Phase Equilibrium. Trans. Chin. Soc. Agric. Eng..

[100] Islam M.M., Hassan M.H., Kalam M.A., Zulkifli N.W.b.M., Habibullah M., Hossain M.M. (2016). Improvement of Cold Flow Properties of Cocos Nucifera and Calophyllum Inophyllum Biodiesel Blends using Polymethyl Acrylate Additive. J. Clean. Prod..

[101] Wang J., Cao L., Han S. (2014). Effect of Polymeric Cold Flow Improvers on Flow Properties of Biodiesel from Waste Cooking Oil. Fuel.

[102] Giraldo S.Y., Rios L.A., Suárez N. (2013). Comparison of Glycerol Ketals, Glycerol Acetates and Branched Alcohol-derived Fatty Esters as Cold-flow Improvers for Palm Biodiesel. Fuel.

[103] Joshi H., Moser B.R., Toler J., Smith W.F., Walker T. (2011). Ethyl levulinate: A Potential Bio-based Diluent for Biodiesel which Improves Cold Flow Properties. Biomass Bioenergy.

[104] Xue Y., Yang C., Xu G., Zhao Z., Lian X., Sheng H., Lin H. (2017). The Influence of Polymethyl Acrylate as a Pour Point Depressant for Biodiesel. Energy Sources Part A Recovery Util. Environ. Eff..

[105] Ballester-Beltrán J., Cantini M., Lebourg M., Rico P., Moratal D., García A.J., Salmerón-Sánchez M. (2012). Effect of Topological cues on Material-driven Fibronectin Fibrillogenesis and Cell Differentiation. J. Mater. Sci. Mater. Med..

[106] Vanterpool F.A., Cantini M., Seib F.P., Salmerón-Sánchez M. (2014). A Material-Based Platform to Modulate Fibronectin Activity and Focal Adhesion Assembly. BioResearch Open Access.

[107] Soria J.M., Martínez Ramos C., Bahamonde O., García Cruz D.M., Salmerón Sánchez M., García Esparza M.A., Casas C., Guzmán M., Navarro X., Gómez Ribelles J.L. (2007). Influence of the Substrate’s Hydrophilicity on the in vitro Schwann Cells Viability. J. Biomed. Mater. Res. Part A.

[108] Wu Y., Ma J., Liu C., Yan H. (2020). Surface Modification Design for Improving the Strength and Water Vapor Permeability of Waterborne Polymer/SiO_2_ Composites: Molecular Simulation and Experimental Analyses. Polymers.

[109] Rahman M.L., Fui C.J., Ting T.X., Sarjadi M.S., Arshad S.E., Musta B. Waste Fiber-Based Cellulose Supported Polymer Ligands for Toxic Metals Removal from Industrial Wastewater. Proceedings of the Presented at the First International Conference on “Green” Polymer Materials.

[110] Rauschendorfer J.E., Vana P. (2021). Increasing the Gas Barrier Properties of Polyethylene Foils by Coating with Poly(methyl acrylate)-Grafted Montmorillonite Nanosheets. Polymers.

[111] Lee C.-J., Wu H., Hu Y., Young M., Wang H., Lynch D., Xu F., Cong H., Cheng G. (2018). Ionic Conductivity of Polyelectrolyte Hydrogels. ACS Appl. Mater. Interfaces.

[112] Lu H., Zhang N., Ma M. (2019). Electroconductive hydrogels for biomedical applications. WIREs Nanomed. Nanobiotechnol..

[113] Kang K., Jung H., An S., Baac H.W., Shin M., Son D. (2021). Skin-like Transparent Polymer-Hydrogel Hybrid Pressure Sensor with Pyramid Microstructures. Polymers.

[114] Wang Z., Wang M., Ma M.-m., Zhang N. (2021). Water-Resistant and Stretchable Conductive Ionic Hydrogel Fibers Reinforced by Carboxymethyl Cellulose. Chin. J. Chem. Phys..

[115] Aziz S.B., Brza M.A., Nofal M.M., Abdulwahid R.T., Hussen S.A., Hussein A.M., Karim W.O. (2020). A Comprehensive Review on Optical Properties of Polymer Electrolytes and Composites. Materials.

[116] Ngai K.S., Ramesh S., Ramesh K., Juan J.C. (2016). A review of polymer electrolytes: Fundamental, approaches and applications. Ionics.

[117] Elliott J.E., Macdonald M., Nie J., Bowman C.N. (2004). Structure and swelling of poly(acrylic acid) hydrogels: Effect of pH, ionic strength, and dilution on the crosslinked polymer structure. Polymer.

[118] Liew C.-W., Ng H.M., Numan A., Ramesh S. (2016). Poly(Acrylic acid)–Based Hybrid Inorganic–Organic Electrolytes Membrane for Electrical Double Layer Capacitors Application. Polymers.

[119] Abu-Saied M., Fahmy A., Morgan N., Qutop W., Abdelbary H., Friedrich J.F. (2019). Enhancement of Poly(vinyl chloride) Electrolyte Membrane by Its Exposure to an Atmospheric Dielectric Barrier Discharge Followed by Grafting with Polyacrylic Acid. Plasma Chem. Plasma Process..

[120] Hosseini S.M., Rahzani B., Asiani H., Khodabakhshi A.R., Hamidi A.R., Madaeni S.S., Moghadassi A.R., Seidypoor A. (2014). Surface modification of heterogeneous cation exchange membranes by simultaneous using polymerization of (acrylic acid-co-methyl methacrylate): Membrane characterization in desalination process. Desalination.

[121] Hosseini S.M., Sohrabnejad S., Nabiyouni G., Jashni E., Van der Bruggen B., Ahmadi A. (2019). Magnetic cation exchange membrane incorporated with cobalt ferrite nanoparticles for chromium ions removal via electrodialysis. J. Membr. Sci..

[122] Khan Y., Bashir S., Hina M., Ramesh S., Ramesh K., Lahiri I. (2020). Effect of Salt Concentration on Poly(Acrylic Acid) Hydrogel Electrolytes and their Applications in Supercapacitor. J. Electrochem. Soc..

[123] Cevik E., Bozkurt A. (2021). Redox active polymer metal chelates for use in flexible symmetrical supercapacitors: Cobalt-containing poly(acrylic acid) polymer electrolytes. J. Energy Chem..

[124] Al Munsur A.Z., Goo B.-H., Kim Y., Kwon O.J., Paek S.Y., Lee S.Y., Kim H.-J., Kim T.-H. (2021). Nafion-Based Proton-Exchange Membranes Built on Cross-Linked Semi-Interpenetrating Polymer Networks between Poly(acrylic acid) and Poly(vinyl alcohol). ACS Appl. Mater. Interfaces.

[125] Isailović J., Vidović K., Hočevar S.B. (2022). Simple electrochemical sensors for highly sensitive detection of gaseous hydrogen peroxide using polyacrylic-acid-based sensing membrane. Sens. Actuators B Chem..

[126] Cheng Y., Hu Y., Xu M., Qin M., Lan W., Huang D., Wei Y., Chen W. (2020). High strength polyvinyl alcohol/polyacrylic acid (PVA/PAA) hydrogel fabricated by Cold-Drawn method for cartilage tissue substitutes. J. Biomater. Sci. Polym. Ed..

[127] Kempson G.E., Muir H., Pollard C., Tuke M. (1973). The tensile properties of the cartilage of human femoral condyles related to the content of collagen and glycosaminoglycans. Biochim. Biophys. Acta (BBA)-Gen. Subj..

[128] Thaitalay P., Thongsri O., Dangviriyakul R., Srisuwan S., Talabnin C., Suksaweang S., Srakaew N.L.-O., Rattanachan S.T. (2021). Influence of polyacrylic acid (PAA)/Na_2_HPO_4_ mixture on biphasic calcium phosphate cement: Enhancing strength and cell viability. Int. J. Appl. Ceram. Technol..

[129] Jiang S., Cao Y., Li S., Pang Y., Sun Z. (2021). Dual function of poly(acrylic acid) on controlling amorphous mediated hydroxyapatite crystallization. J. Cryst. Growth.

[130] Lemanowicz M., Mielańczyk A., Walica T., Kotek M., Gierczycki A. (2021). Application of Polymers as a Tool in Crystallization—A Review. Polymers.

[131] Wang Y., Wang J., Yuan Z., Han H., Li T., Li L., Guo X. (2017). Chitosan cross-linked poly(acrylic acid) hydrogels: Drug release control and mechanism. Colloids Surf. B Biointerfaces.

[132] Soliman M.M., Sakr T.M., Rashed H.M., Hamed A.A., Abd El-Rehim H.A. (2021). Polyethylene oxide–polyacrylic acid–folic acid (PEO-PAAc) nanogel as a ^99m^Tc targeting receptor for cancer diagnostic imaging. J. Label. Compd. Radiopharm..

[133] Chiong J.A., Tran H., Lin Y., Zheng Y., Bao Z. (2021). Integrating Emerging Polymer Chemistries for the Advancement of Recyclable, Biodegradable, and Biocompatible Electronics. Adv. Sci..

[134] Coates G.W., Getzler Y.D.Y.L. (2020). Chemical recycling to monomer for an ideal, circular polymer economy. Nat. Rev. Mater..

[135] Bin Rusayyis M., Torkelson J.M. (2020). Recyclable Polymethacrylate Networks Containing Dynamic Dialkylamino Disulfide Linkages and Exhibiting Full Property Recovery. Macromolecules.

[136] Dubois J.-L. New Innovative process for recycling end-of-life PMMA waste. MMAtwo second generation Methyl Methacrylate. Proceedings of the Conference Plastic Recycling Technologies.

[137] Innovative Acrylic(PMMA-Polymethyl Methacrylate) Recycling Technology Complying with Regulations. https://cordis.europa.eu/project/id/856103.

[138] Post W., Susa A., Blaauw R., Molenveld K., Knoop R.J.I. (2020). A Review on the Potential and Limitations of Recyclable Thermosets for Structural Applications. Polym. Rev..

[139] Sudhakar Y.N., Selvakumar M., Bhat D.K. (2021). Enhancement and Investigation of Biodegradability of Poly(Methyl Methacrylate) and Poly(Vinyl Chloride) by Blending with Cellulose Derivatives.

[140] Hou Q., Zhen M., Qian H., Nie Y., Bai X., Xia T., Laiq Ur Rehman M., Li Q., Ju M. (2021). Upcycling and catalytic degradation of plastic wastes. Cell Rep. Phys. Sci..

[141] Jeon C., Kim D.W., Chang S., Kim J.G., Seo M. (2019). Synthesis of Polypropylene via Catalytic Deoxygenation of Poly(methyl acrylate). ACS Macro Lett..

[142] Rathee V.S., Sidky H., Sikora B.J., Whitmer J.K. (2018). Role of Associative Charging in the Entropy–Energy Balance of Polyelectrolyte Complexes. J. Am. Chem. Soc..

[143] Raczkowska J., Stetsyshyn Y., Awsiuk K., Lekka M., Marzec M., Harhay K., Ohar H., Ostapiv D., Sharan M., Yaremchuk I. (2017). Temperature-responsive grafted polymer brushes obtained from renewable sources with potential application as substrates for tissue engineering. Appl. Surf. Sci..

[144] Yu D., Zhao J., Wang W., Qi J., Hu Y. (2019). Mono-acrylated isosorbide as a bio-based monomer for the improvement of thermal and mechanical properties of poly(methyl methacrylate). RSC Adv..

[145] Veith C., Diot-Néant F., Miller S.A., Allais F. (2020). Synthesis and polymerization of bio-based acrylates: A review. Polym. Chem..

[146] Stetsyshyn Y., Raczkowska J., Budkowski A., Awsiuk K., Kostruba A., Nastyshyn S., Harhay K., Lychkovskyy E., Ohar H., Nastishin Y. (2016). Cholesterol-Based Grafted Polymer Brushes as Alignment Coating with Temperature-Tuned Anchoring for Nematic Liquid Crystals. Langmuir.

